# Extrinsic apoptosis and necroptosis in telencephalic development: a single-cell mass cytometry study

**DOI:** 10.1038/s41418-025-01594-5

**Published:** 2025-10-21

**Authors:** Jiachen Shi, Weile Liu, Alison Song, Timi Sanni, Amy Van Deusen, Eli R. Zunder, Christopher D. Deppmann

**Affiliations:** 1 Department of Biology, College of Arts and Sciences, University of Virginia, Charlottesville, VA USA; 2 Neuroscience Graduate Program, School of Medicine, University of Virginia, Charlottesville, VA USA; 3 Department of Biomedical Engineering, School of Engineering, University of Virginia, Charlottesville, VA USA; 4 Program in Fundamental Neuroscience, College of Arts and Sciences, University of Virginia, Charlottesville, VA USA; 5 Department of Neuroscience, School of Medicine, University of Virginia, Charlottesville, VA USA; 6 Department of Cell Biology, School of Medicine, University of Virginia, Charlottesville, VA USA

**Keywords:** Neuroscience, Cell biology

## Abstract

Regulated cell death is integral to sculpting the developing brain, yet the relative contributions of extrinsic apoptosis and necroptosis remain unclear. Here, we leverage single-cell mass cytometry (CyTOF) to characterize the cellular landscape of the mouse telencephalon in wild-type (WT), RIPK3 knockout (RIPK3 KO), and RIPK3/Caspase-8 double knockout (DKO) mice. Strikingly, combined deletion of RIPK3 and Caspase-8 leads to a 12.6% increase in total cell count, challenging the prevailing notion that intrinsic apoptosis exclusively governs developmental cell elimination. Detailed subpopulation analysis reveals that DKO mice display selective enrichment of Tbr2⁺ intermediate progenitors and endothelial cells, underscoring distinct, cell type–specific roles for extrinsic apoptotic and necroptotic pathways. These findings provide a revised framework for understanding the coordinated regulation of cell number during telencephalic development and suggest potential mechanistic links to neurodevelopmental disorders characterized by aberrant cell death.

## Introduction

During early development, the central nervous system (CNS) generates an excess of neurons and glia, subsequently eliminating up to 50% to ensure proper circuit formation and brain function [1–3]. Regulation of cell number for specific cell types during this process is orchestrated by a combination of factors: cell proliferation, cell differentiation, and regulated cell death. Historically, intrinsic (mitochondrial) apoptosis was considered the primary mechanism driving developmental cell death, a view supported by abundant cleaved Caspase-3 and TUNEL-positive cells [4–6], as well as by the severe malformations observed in mice lacking key intrinsic apoptotic components such as Bax/Bak, Caspase-9, or Apaf-1 [7–10]. However, since both cleaved Caspase-3 and TUNEL positivity can result from death pathways including extrinsic apoptosis, these classical markers alone cannot definitively attribute developmental cell elimination solely to intrinsic apoptosis. Furthermore, even in mice lacking the key intrinsic apoptotic components Apaf-1^−/−^ and Bax^−/−^Bak^−/−^, substantial cell death persists in multiple tissues [11, 12], including interdigital cell death, suggesting the involvement of alternative mechanisms such as extrinsic apoptosis.

To genetically dissect the contribution of extrinsic apoptosis to cell elimination during neural development, the initiator Caspase-8 is an intuitive target because it serves as the convergence point for multiple death receptors such as Fas/CD95, TRAILR1, and TNFR1 [13–15]. Upon death receptor activation, procaspase-8 and adaptor protein FADD are recruited to form the death-inducing signaling complex (DISC). DISC assembly promotes procaspase-8 dimerization and autoprocessing to active Caspase-8, which then triggers apoptosis by cleaving and activating executioner Caspase-3 [15, 16]. Beyond direct caspase activation, Caspase-8 also amplifies the death signal by generating truncated Bid (tBid), which promotes mitochondrial outer membrane permeabilization (MOMP) through both Bax/Bak-dependent and independent mechanisms [17–19]. Given its central role, Caspase-8 would seem to be a straightforward target for studying extrinsic apoptosis in neural development. However, Caspase-8 also negatively regulates other cell death pathways. Consequently, its loss not only disrupts extrinsic apoptosis but also serves as a gain-of-function for alternative death mechanisms, complicating the interpretation of the knockout phenotype.

In addition to its canonical role mediating extrinsic apoptosis, Caspase-8 activity also suppresses necroptosis by inhibiting activation of the RIPK1-RIPK3-MLKL signaling cascade [20–24]. The consequence of disrupting this dual function is severe: Caspase-8-deficient mice die embryonically by E11.5 due to unopposed necroptotic signaling, which gives rise to hyperaemia, neural tube undulation, and vascular defects/dysfunction [25, 26]. Notably, the vascular phenotype appears central to this lethality, as conditional knockout of Caspase-8 in endothelial cells (Casp8^F/−^Tie1-Cre) recapitulates the vascular defects and embryonic lethality of the global knockout, suggesting endothelial cells as a key population vulnerable to necroptotic cell death [27]. The role of necroptosis is further confirmed by the rescue of embryonic lethality through concurrent deletion of RIPK3, demonstrating the critical importance of this regulatory balance during development [23, 26].

Collectively, these findings not only underscore the centrality of extrinsic apoptosis in neural development but also highlight necroptosis as a mechanistically distinct pathway with significant developmental implications. Unchecked activation of the RIPK1-RIPK3-MLKL cascade, as observed in Caspase-8–deficient contexts, suggests that necroptosis contributes to vascular homeostasis during embryogenesis. Moreover, the potential for necroptosis to modulate cell numbers—complementing the cell elimination mediated by apoptotic pathways—provides a compelling rationale for its investigation as an additional regulatory mechanism that fine-tunes tissue architecture during telencephalic development.

To systematically investigate the contributions of both extrinsic apoptosis and necroptosis during development, we employed a comparative genetic approach using RIPK3/Caspase-8 double knockout (DKO) mice alongside RIPK3 single knockouts (RIPK3 KO) and wild-type (WT) controls. This experimental design creates a powerful framework to examine both pathways: comparing DKO with RIPK3 KO phenotypes reveals the specific contributions of Caspase-8-mediated extrinsic apoptosis, while comparing RIPK3 KO with wild-type mice illuminates the role of RIPK3-mediated necroptosis. This strategy not only circumvents the necroptosis-mediated embryonic lethality associated with isolated Caspase-8 deletion, but also allows us to examine how these interconnected death pathways operate in parallel during neural development.

To investigate the effect of these knockouts on cell number regulation during neural development, we applied single-cell mass cytometry (CyTOF) measurement to simultaneously quantify multiple protein markers, including cleaved Caspase-3, across diverse cell populations [28–30]. We focused on the telencephalon as our model system due to its well-characterized extensive cellular remodeling and neural-vascular interactions during development [31–34]. Here, we leverage CyTOF in conjunction with genetic models (DKO, RIPK3 KO, and WT) to dissect how different modes of regulated cell death shape telencephalic development. Our goals are to (1) map the distribution of extrinsic apoptosis and necroptosis across distinct cellular populations; (2) identify compensatory or synergistic interactions among death pathways; and (3) determine how these processes interface with neurovascular development. Through these investigations, we reveal new cell type-specific roles for extrinsic apoptosis and necroptosis and show that the combined loss of RIPK3 and Caspase-8 significantly increases telencephalic cell number—highlighting the previously underappreciated importance of death receptor signaling in neurodevelopment. Our findings establish a framework for understanding how cell death pathways work together to refine brain architecture and may have implications for therapies targeting aberrant cell death in neurodevelopmental disorders [35, 36].

## Results

### Profiling cell death and proliferation in developing telencephalon neural cell types

**Fig. 1:**
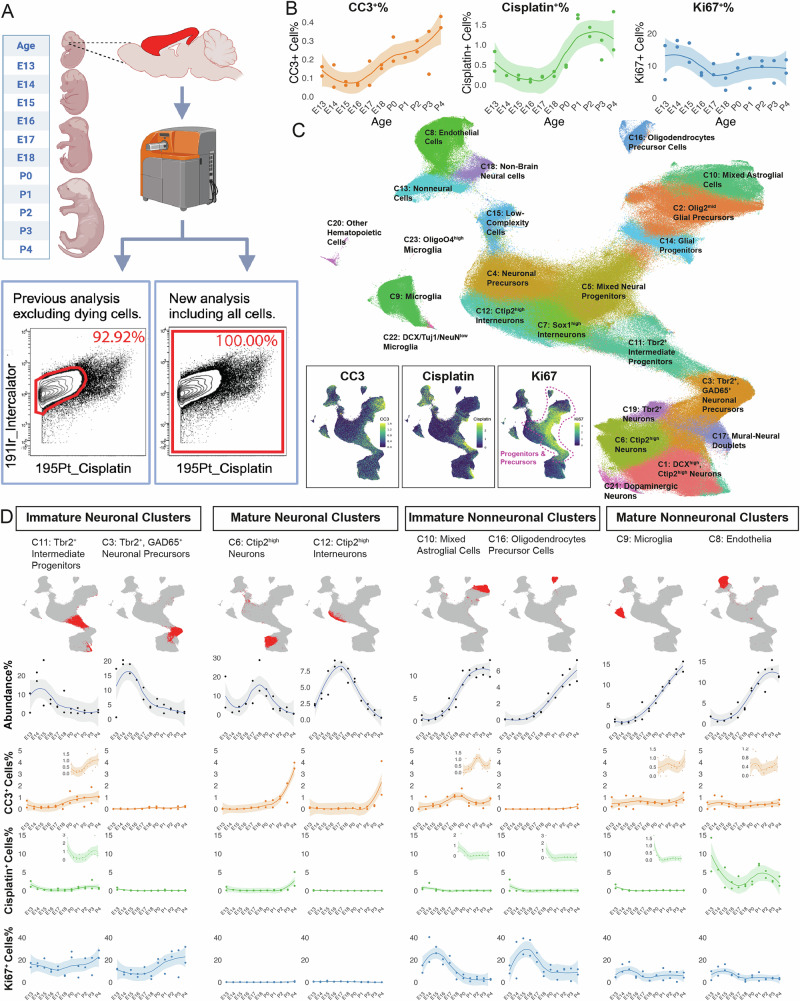
Cell death, proliferation, and abundance in the developing telencephalon. **A** Schematic representation of the modified experimental workflow for including both live and dying cells. Single-cell mass cytometry data from mouse telencephalon (E13-P4) were reanalyzed to include both live and dying cells. Flow cytometry plots show the inclusion of Cisplatin^+^ cells in the new analysis. **B** Temporal dynamics of CC3^+^%, Cisplatin^+^%, and Ki67^+^% cells throughout development (E13-P4). Individual replicates are shown along with the Loess curve fitting of the data. **C** UMAP visualization of 23 distinct cell clusters identified by Leiden clustering. Insets show the expression patterns of CC3 (apoptosis), Cisplatin (plasma membrane integrity), and Ki67 (proliferation) across the UMAP layout. Cell clusters identified as precursor and progenitor cell types by their protein expression profiles are highlighted by a red dashed line in the Ki67 inset. **D** Detailed analysis of selected clusters representing immature neuronal, mature neuronal, immature nonneuronal, and mature nonneuronal cell populations. Top: UMAP plots highlighting the distribution of each cluster. Bottom: Temporal dynamics of cluster abundance% (gray), CC3^+^ cells% (orange), Cisplatin^+^ cells% (green), and Ki67^+^ cells% (blue) from E13 to P4. Individual replicates are shown along with Loess curve fitting of the data.

To systematically characterize the temporal dynamics of cellular proliferation and death during mouse brain development, we performed a comprehensive re-analysis of single-cell mass cytometry data from our previously published developmental atlas of the CNS with daily timepoints from E11 to P4 [29]. To include dying cell populations that were excluded from our previous analysis, we removed the Cisplatin-based viability gate [37] from our analysis pipeline (Fig. 1A). This approach allowed simultaneous quantification of three distinct cellular states using key markers: Ki67 for proliferation [38, 39], cleaved Caspase-3 (CC3) for apoptosis [4, 40], and Cisplatin for plasma membrane integrity [37]. It is important to note that while Cisplatin is used in clinical settings as a chemotherapeutic agent that enters cells and forms DNA adducts, which can trigger apoptosis [37, 41], this process takes place on the hour time scale, and our protocol’s viability stain with Cisplatin only lasts for 30 s. This short exposure followed by immediate PFA fixation (Methods) ensures that Cisplatin doesn’t induce apoptosis in our cell samples, instead only acting as a viability dye via elevated staining levels in dying cells with compromised plasma membranes [37].

Re-analysis including all dead and dying cells in the mouse telencephalon revealed that global cell death progressively increased from E13 to P4, as evidenced by elevated percentages of both CC3^+^ and Cisplatin^+^ cells (Fig. 1B). Over this time period, CC3^+^ cells increased by 203.0% and Cisplatin^+^ cells by 129.9%, with a significant rise in marker-positive cells across developmental age (linear regression: *p* = 6.0 × 10⁻⁵, R² = 0.561 for CC3^+^%; *p* = 0.0015, R² = 0.435 for Cisplatin^+^%). This temporal pattern aligns with established developmental cell death dynamics, where a major wave of apoptosis occurs during the first postnatal week, eliminating up to 30% of neurons [42, 43]. Previous studies have demonstrated that neocortical cell death exhibits characteristic temporal dynamics: initiating at P2, reaching maximal levels during P4-P7, followed by gradual reduction through P9-P10 [44].

In addition to these temporal changes, we identified distinct cell populations exhibiting specific combinations of death markers (Supplementary Fig. S1A): CC3^+^Cisplatin^−^ cells, CC3^−^Cisplatin^+^ cells, and CC3^+^Cisplatin^+^ cells. Based on established cell death mechanisms, where classical apoptosis typically maintains membrane integrity while non-apoptotic pathways such as necroptosis are characterized by early membrane rupture [45], these populations were interpreted as follows: CC3^+^Cisplatin^−^ cells represent early apoptotic cells with intact membrane integrity; CC3^−^Cisplatin^+^ events suggest cells undergoing non-apoptotic death with compromised membranes; and CC3^+^Cisplatin^+^ cells potentially indicate a combination of later-stage apoptotic and non-apoptotic death [45, 46]. This heterogeneity in death marker profiles hints at an interplay between different cell death mechanisms during development.

While cell death showed temporal progression, we did not observe a clear pattern in proliferation (Fig. 1B). The percentage of Ki67^+^ ranges from 2.35% to 17.77% across developmental stages, without a simple trend (linear regression: *p* = 0.144, R² = 0.103). This complex pattern at the population level likely reflects the partially overlapping waves of neurogenesis (E12 through late embryonic stage) and gliogenesis (late embryonic stage through early postnatal stage) [47–49], during which proliferative activity transitions between different progenitor populations. To uncover cell type-specific patterns that might be masked by these population averages, we next performed detailed analyses of distinct cell populations.

To characterize distinct cell populations, we performed Leiden clustering [50] and visualized the results using Uniform Manifold Approximation and Projection (UMAP) [51]. The analysis revealed 23 distinct clusters (Fig. 1C, Supplementary Fig. S1B), which were subsequently grouped into four broad categories based on their unique protein expression profiles: 1) immature neuronal clusters, 2) mature neuronal clusters, 3) immature nonneuronal clusters, and 4) mature nonneuronal clusters. This classification was established using differential expression patterns of validated markers for neuronal maturity (e.g., PAX6, Tbr2, DCX, Tbr1, Ctip2) [52–54] and glial cell types (e.g., GFAP, Olig2, CD11b, PECAM) [55–57].

Analysis of proliferation and death markers across the UMAP layout revealed distinct maturation-associated patterns (Fig. 1C insets). Ki67 exhibited high intensity in progenitor and precursor clusters, particularly in mixed neural progenitors, glial progenitors, and Tbr2^+^ intermediate progenitors. This enrichment of the proliferation marker in progenitor and precursor populations aligns with established patterns of active cell division in neural progenitor cells during CNS development [58]. Conversely, CC3 and Cisplatin showed higher intensity in more differentiated populations, notably in mixed astroglial cells and endothelial cells.

Detailed temporal analysis of individual clusters revealed distinct death and proliferation patterns in neuronal populations (Fig. 1D, Supplementary Fig. S1C). Most immature neuronal clusters maintained high percentages of Ki67^+^ cells ( > 10%) while showing low percentages of both CC3^+^ and Cisplatin^+^ cells (0–2%). In contrast, mature neuronal clusters generally exhibited limited proliferation (Ki67^+^ cells <2%) and low death marker expression (CC3^+^ and Cisplatin^+^ cells 0–2%), although there were some notable exceptions. Specifically, Ctip2^high^ neurons and Ctip2^high^ interneurons showed progressive increases in apoptotic cells, with CC3^+^% rising from 0% at E13.5 to around 4% at P4 (linear regression: *p* = 9.8 × 10⁻⁵, R² = 0.540 for Ctip2^high^ neurons; *p* = 0.028, R² = 0.220 for Ctip2^high^ interneurons). While mature neurons generally exhibit higher resistance to death signals in the adult brain [59], our analysis window (E13 to P4) includes a critical period of developmental apoptosis (P0-P4) when neurons are particularly susceptible to death signals as circuits are refined and excess cells are eliminated [42, 43, 60].

Nonneuronal clusters exhibited more complex patterns (Fig. 1D, Supplementary Fig. S2). The relative abundance of most nonneuronal clusters started to increase around E15–E18, with astrogenesis starting around E16–E17. This timing aligns with the established developmental program where gliogenesis follows neurogenesis, with astrocyte production beginning in late embryonic stages and continuing through early postnatal development [47]. While immature nonneuronal clusters generally maintained high percentages of Ki67^+^ cells ( > 15%), some mature nonneuronal clusters also displayed significant Ki67^+^ expression (5-20%), consistent with the established pattern where glial cells, unlike neurons, retain proliferative capacity throughout development [61, 62]. Notably, specific immature nonneuronal clusters (e.g., Olig2^med^ Glial Precursors, Astroglial Precursors, and Oligodendrocyte Precursor Cells) showed a distinct temporal pattern of proliferation, characterized by Ki67^+^% increasing from ~40% at E13 to a peak of ~70% around E15-E17 (linear regression: *p* = 0.011, R² = 0.577 for Olig2^med^ Glial Precursors from E13 to E17; *p* = 0.16, R² = 0.426 for Astroglial Precursors from E13 to E15; *p* = 0.11, R² = 0.370 for Oligodendrocyte Precursor Cells from E13 to E16), followed by a subsequent decrease to ~30% by P4 (linear regression: *p* = 0.020, R² = 0.373 for Olig2^med^ Glial Precursors from E17 to P4; *p* = 8.1 × 10⁻^6^, R² = 0.722 for Astroglial Precursors from E15 to P4; *p* = 0.0046, R² = 0.447 for Oligodendrocyte Precursor Cells from E16 to P4).

In terms of cell death dynamics, mature nonneuronal clusters displayed varying levels of death markers. Notably, endothelial cells demonstrated an intriguing discrepancy between markers: while Cisplatin uptake was substantial (5–15% of cells), cleaved Caspase-3 (CC3) expression remained relatively low ( < 2%). The high Cisplatin positivity coupled with minimal CC3 activation indicates the engagement of alternative cell death pathways such as necroptosis in developmental vascular pruning. Though CC3 analysis captured both intrinsic and extrinsic apoptotic pathways, and Cisplatin indicated potential necroptotic death, the unexpected divergence between these markers prompted us to investigate specific death pathways through genetic perturbation of extrinsic apoptosis and necroptosis.

### Expression of extrinsic apoptosis and necroptosis components in the telencephalon

**Fig. 2:**
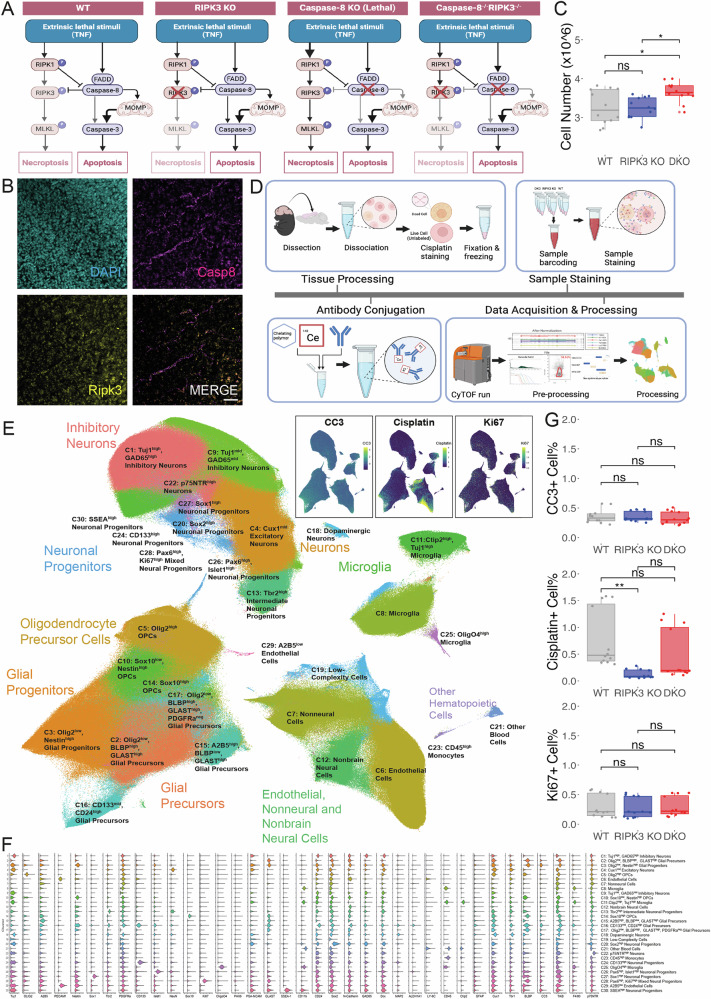
Investigation of extrinsic apoptosis and necroptosis in telencephalon development. **A** Schematic representation of extrinsic apoptotic and necroptotic pathways in WT, RIPK3 KO, Caspase-8 KO, and DKO mice. **B** Representative images of RNAscope in situ hybridization for Casp8 (magenta) and Ripk3 (yellow) mRNA in P4 WT mouse cortex. DAPI (cyan) labels nuclei. Scale bar: 50 μm. **C** Quantification of total cell numbers in the telencephalon of P4 mice (*n* = 12 per genotype). **D** Workflow schematic for mass cytometry analysis of P4 mouse telencephalon from WT, RIPK3 KO, and DKO mice, including tissue processing, antibody conjugation, sample staining, and data acquisition and processing steps. **E** UMAP visualization of mass cytometry data showing 30 distinct cell clusters identified by Leiden clustering. Major cell type categories are annotated. Insets show expression patterns of CC3, Cisplatin, and Ki67 across the UMAP layout. **F** Violin plot showing relative expression of all 37 markers across the 30 identified cell clusters. **G** Quantification of CC3^+^, Cisplatin^+^, and Ki67^+^ cells% across genotypes from mass cytometry data. For (**C**, **G**), data are presented as box plots where the middle line represents the median, the box represents the interquartile range (IQR), and the whiskers extend to 1.5 times the IQR. Individual data points are shown as dots. Statistical differences between groups are denoted by asterisks. Statistical significance levels are as follows: *p* < *0.05*, *p < 0.01, ***p < 0.001, ns not significant (one-way ANOVA with Tukey’s post-hoc test).

Extrinsic apoptosis and necroptosis act through distinct but intersecting modules centered on Caspase-8 and RIPK3, respectively (Fig. 2A). To investigate the contribution of these pathways to developmental cell death in vivo, we leveraged a comparative genetic approach using RIPK3/Caspase-8 double-knockout (DKO) mice, alongside RIPK3 single knockouts (RIPK3 KO) and wild-type (WT) controls. This design enables functional dissection of pathway-specific contributions: comparison of DKO and RIPK3 KO phenotypes isolates Caspase-8–dependent effects, while RIPK3 KO vs. WT highlights necroptosis-specific mechanisms. Importantly, this approach circumvents the embryonic lethality caused by Caspase-8 deletion alone, allowing us to study both pathways in parallel during brain development.

We initially attempted to detect pathway-specific proteins - cleaved Caspase-8 (CC8) for extrinsic apoptosis and phosphorylated MLKL (pMLKL) for necroptosis - via immunohistochemistry (IHC). However, extensive testing of multiple commercial antibodies revealed a lack of specificity, as demonstrated by inconsistent staining patterns and high background signal (Supplementary Fig. S4 and S5). Given the strong developmental regulation of death receptors and their ligands, with peak expression at P5, and minimal detection in adult brain [63], we hypothesized that analyzing mRNA expression patterns might reveal regulatory mechanisms governing apoptotic and necroptotic pathways. Using RNAscope to examine P4 WT mouse cortex (*n* = 3), we observed widespread Casp8 mRNA expression throughout the tissue. Quantitative colocalization analysis revealed that 73.0 ± 13.6% (mean ± SD) of RIPK3 mRNA puncta exhibited expression that coincided with Casp8 mRNA signals (Fig. 2B, Supplementary Fig. S6A). This pattern was specific to developing brains, as no similar colocalization was observed in adult cortex (Supplementary Fig. S6B). The co-expression of these death pathway components resembles endothelial cells, which is particularly intriguing given that endothelial disorganization and subsequent circulatory failure have been identified as the primary cause of embryonic lethality in Caspase-8 knockout mice [26, 27]. This spatial convergence suggests potential crosstalk between these death mechanisms in endothelial cells.

To functionally assess whether these transcriptional patterns translate into downstream activation of extrinsic apoptosis and necroptosis signaling, we next used CyTOF to analyze the telencephalon of RIPK3 KO, DKO, and WT mice at P4. This time point was selected for two strategic reasons: first, this represents a critical window to capture the cumulative effects of both late embryonic and early postnatal waves of cell death [42, 44]; second, this time point allows optimal preparation of high-quality single-cell suspensions for downstream CyTOF analysis [29, 64].

### Impact of death receptor pathway deletions on telencephalic cell number

Initial phenotypic characterization using automated cell counting of telencephalon revealed no significant difference in total cell count between RIPK3 KO (3.25 ± 0.27 × 10^6^ cells) and WT groups (3.27 ± 0.44 × 10^6^ cells; *p* = 0.986). However, the DKO group exhibited a significantly higher cell count (3.66 ± 0.26 × 10^6^ cells) compared to both WT and RIPK3 KO groups (*p* < 0.05 for both comparisons) (Fig. 2C). This effect was observed in both male and female mice, with no significant sex-by-genotype interaction (Supplementary Fig. S7). Cell counts were measured for individual pups (*n* = 12 per genotype, 6 males and 6 females), drawn from three independent litters per genotype. The significant increase in cell number specifically in DKO mice (12-13% higher than both WT and RIPK3 KO, *p* < 0.05), coupled with the lack of effect in RIPK3 single knockouts, suggests that Caspase-8-mediated extrinsic apoptosis significantly contributes to cell elimination during telencephalon development. While we cannot completely exclude synergistic effects or compensatory mechanisms triggered by combined pathway deletion, these findings establish death receptor signaling as a key regulator of developmental cell death.

To elucidate the cellular mechanisms underlying the observed increases in cell number, we performed high-dimensional mass cytometry using a 37-antibody panel (Fig. 2D, Supplementary Table 1, Supplementary Fig. S8) on 3,604,163 single cells from the developing telencephalon. Initial Leiden clustering identified 30 distinct clusters (Fig. 2E, F), with UMAP visualization showing clear segregation of major cell populations based on established markers [29] (Supplementary Fig. S9): neurons (NeuN^+^, MAP2^+^, TuJ1^+^), astrocytes (GFAP^+^, ALDH1A1^+^), oligodendrocytes (Olig2^+^, OligO4^+^, Sox10^+^), microglia (CD45^+^, CD11b^+^), and endothelial cells (CD31^+^, Ly6C^+^). To achieve higher resolution, we performed subclustering analysis on four major cellular categories: Neuronal populations; Glial precursor populations; Endothelial, Astrocytic, and Nonneural populations; and Microglial, Blood, and ﻿﻿MonoMonocytetyp﻿e﻿ populations. This refined analysis revealed 89 distinct subpopulations (Supplementary Fig. S10).

Analysis of cell death markers revealed three key findings (Fig. 2G): (1) Classic apoptotic markers remained unchanged across genotypes (CC3^+^ cells: WT 0.358 ± 0.116%, RIPK3 KO 0.355 ± 0.084%, DKO 0.329 ± 0.106%; *p* > 0.05). (2) Proliferation rates showed no significant differences (Ki67^+^ cells: WT 0.298 ± 0.195%, RIPK3 KO 0.272 ± 0.170%, DKO 0.293 ± 0.166%; *p* > 0.05). (3) Membrane permeability showed striking genotype-dependent effects with RIPK3 KO mice exhibiting significantly reduced Cisplatin uptake compared to WT (0.125 ± 0.082% vs 0.788 ± 0.552%, *p* < 0.01) and DKO mice showing intermediate levels (0.481 ± 0.481%), suggesting compensatory mechanisms that partially restore membrane permeability [65]. While this global analysis reveals intriguing population-level changes across genotypes, definitive determination of cell type-specific roles for these death pathways requires higher resolution analysis of each cellular compartment.

To explore cell type-specific changes, we started with hierarchical clustering analysis of marker expression across 30 initial clusters, revealing distinct molecular signatures associated with genotype (Supplementary Fig. S11). The resulting heatmap demonstrates clear segregation of cellular clusters based on lineage-specific markers. Cross-genotype comparisons revealed significant population changes, particularly between DKO vs. WT and RIPK3 KO vs. WT, as shown in the *p*-value heatmap (Supplementary Fig. S11, right panels). These changes were especially pronounced in glial and non-neuronal populations, including microglia (C8, C25), endothelial cells (C6), low-complexity cells (C19), nonbrain cells (C12), and blood cells (C21), suggesting cell type-specific regulation by extrinsic apoptosis and necroptosis.

### Selective expansion or depletion of neuronal populations following death receptor pathway disruption

**Fig. 3:**
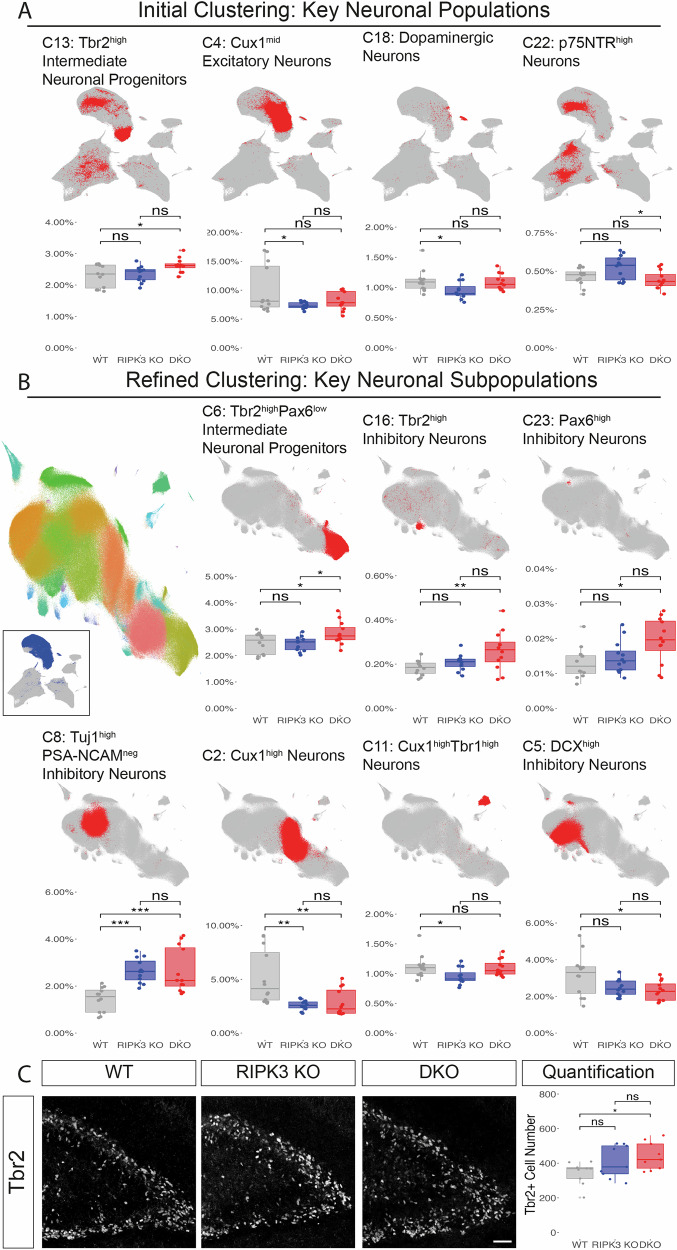
Differential abundance of telencephalic neuronal subpopulations. **A** Initial clustering analysis showing UMAP visualization and quantification of four major neuronal populations. For each cluster, relative abundance% is compared across WT, RIPK3 KO, and DKO. **B** Refined clustering analysis showing UMAP visualization and quantification of key neuronal subpopulations. Left UMAP plot displays 26 distinct neuronal subclusters (C1–C26) identified through iterative Leiden clustering (colored overlay), with inset showing the analyzed clusters from initial clustering (dark blue). Representative UMAP plots and quantification are shown for seven key neuronal populations. For each cluster, relative abundance% is compared across WT, RIPK3 KO, and DKO. **C** IHC validation of Tbr2^+^ cells. Left: Representative images of Tbr2 staining in the dentate gyrus across genotypes. Right: Quantification of Tbr2^+^ cell numbers in the dentate gyrus. Scale bar: 50 μm. For all quantifications: Data are presented as box plots showing median (middle line), interquartile range (box), and whiskers (1.5 × IQR), with individual data points overlaid. Statistical significance determined by one-way ANOVA with Tukey’s post-hoc test (*p < 0.05, **p < 0.01, ***p < 0.001, ns: not significant).

Given these broad population changes, we next performed a detailed characterization of specific cellular compartments. Comprehensive analysis of neuronal populations across genotypes revealed distinct alterations in both progenitor and mature neuronal compartments, with particularly striking differences in intermediate progenitor populations. Initial clustering analysis identified four key neuronal populations with genotype-specific abundance patterns (Fig. 3A): Tbr2^high^ Intermediate Neuronal Progenitors (C13), Cux1^mid^ Excitatory Neurons (C4), Dopaminergic Neurons (C18), and p75NTR^high^ Neurons (C22). Among these, DKO mice exhibited a selective increase in Tbr2^high^ Intermediate Neuronal Progenitors (2.63 ± 0.22%) compared to both WT (2.30 ± 0.36%, *p* < 0.05) and RIPK3 KO (2.45 ± 0.27%) populations.

Further subclustering of neuronal populations revealed more nuanced genotype-dependent differences (Fig. 3B). Tbr2^high^Pax6^low^ Intermediate Neuronal Progenitors (C6) showed significant enrichment in DKO mice (2.84 ± 0.43%) compared to both WT (2.46 ± 0.39%, *p* < 0.05) and RIPK3 KO (2.45 ± 0.27%, *p* < 0.05). Similarly, Tbr2^high^ Inhibitory Neuron population (C16) exhibited increased abundance in DKO mice (0.26 ± 0.09%) versus WT controls (0.18 ± 0.03%, *p* < 0.01). We also observed elevated proportions of Pax6^high^ Inhibitory Neurons (C23) in DKO mice (0.019 ± 0.0065%) compared to WT controls (0.013 ± 0.0046%, *p* < 0.05). Investigation of mature neuronal populations revealed additional genotype-specific differences. Both RIPK3 KO (2.65 ± 0.51%) and DKO (2.66 ± 0.95%) mice showed significant increases in Tuj1^high^PSA-NCAM^neg^ Inhibitory Neurons (C8) compared to WT controls (1.43 ± 0.52%, *p* < 0.001). Conversely, Cux1^high^ neurons (C2) decreased in both RIPK3 KO (2.60 ± 0.51%) and DKO mice (2.85 ± 0.51%) compared to WT controls (5.17 ± 2.52%, *p* < 0.01). Additionally, Cux1^high^Tbr1^high^ Neurons (C11) were significantly reduced in RIPK3 KO mice (0.95 ± 0.14%) compared to WT (1.12 ± 0.19%, *p* < 0.05), while DKO mice exhibited decreased DCX^high^ inhibitory neurons (C5) (1.12 ± 0.31% vs WT: 1.85 ± 0.42%, *p* < 0.05).

To validate the mass cytometry findings, we performed IHC analysis of Tbr2^+^ cells in the dentate gyrus (Fig. 3C). This orthogonal approach confirmed increased Tbr2^+^ cell numbers in DKO mice (441 ± 38 cells) compared to WT controls (343 ± 29 cells, *p* < 0.05), with RIPK3 KO mice displaying an intermediate phenotype (372 ± 33 cells) not significantly different from either WT or DKO (*p* > 0.05).

### Caspase-8 is required for restricting neurovascular and OPC cell number

**Fig. 4:**
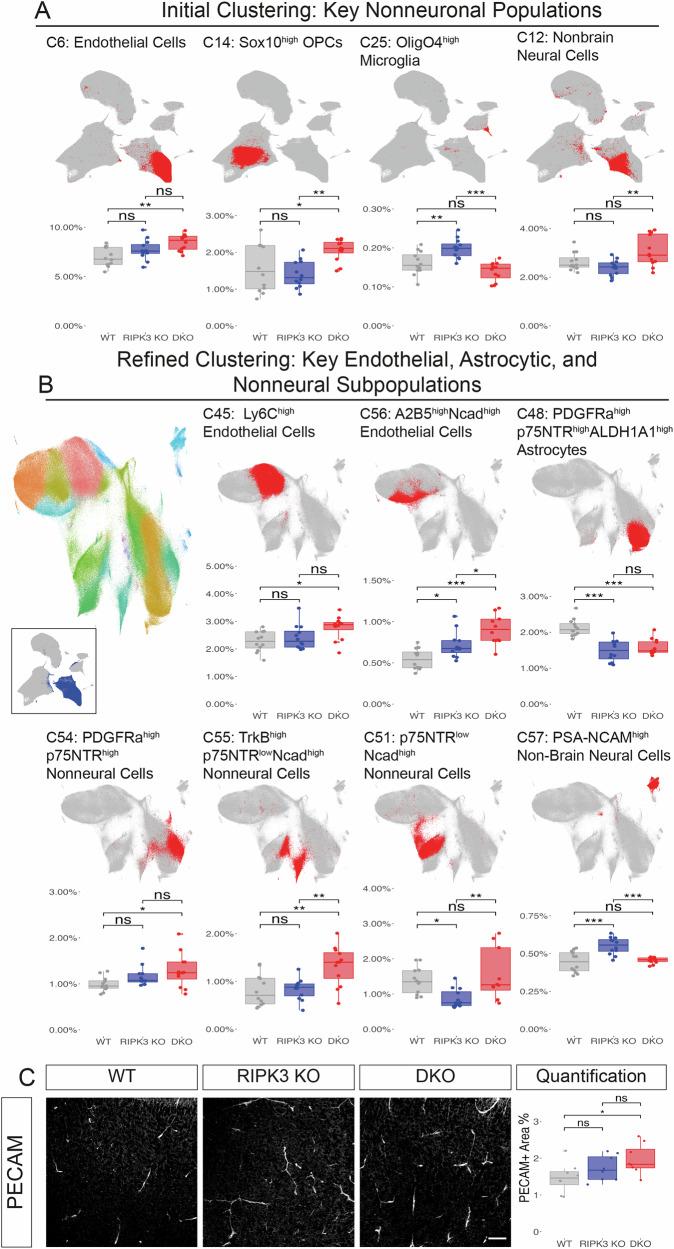
Differential abundance of telencephalic nonneuronal subpopulations. **A** Initial clustering analysis showing UMAP visualization and quantification of four major nonneuronal populations. For each cluster, relative abundance% is compared across WT, RIPK3 KO, and DKO. **B** Refined clustering analysis showing UMAP visualization and quantification of key endothelial, astrocytic, and nonneural subpopulations. Left UMAP plot displays 22 distinct nonneuronal subclusters (C45-C66) identified through iterative Leiden clustering (colored overlay), with inset showing the analyzed clusters from initial clustering (dark blue). Representative UMAP plots and quantification are shown for seven key endothelial, astrocytic, and nonneural subpopulations. For each cluster, relative abundance% is compared across WT, RIPK3 KO, and DKO. **C** IHC validation of PECAM+ area. Left: Representative images of PECAM staining in the cortex across genotypes. Right: Quantification of PECAM+ Area% in the cortex. Scale bar: 50 μm.

Analysis of nonneuronal populations revealed striking genotype-dependent alterations in cellular composition, with particularly dramatic effects on vascular and glial lineages. Initial clustering analysis identified four distinct nonneuronal populations exhibiting genotype-specific abundance patterns (Fig. 4A): Endothelial Cells (C6), Sox10^high^ OPCs (C14), OligO4^high^ microglia (C25), and Nonbrain Neural Cells (C12). The most pronounced differences emerged in the endothelial compartment, where DKO mice displayed significantly elevated populations (8.45 ± 0.83%) compared to WT controls (6.95 ± 0.96%, *p* < 0.01). (Fig. 4A). This vascular expansion was accompanied by marked changes in oligodendrocyte lineage cells, with Sox10^high^ OPCs showing significant enrichment in DKO mice (2.06 ± 0.30%) relative to both WT (1.58 ± 0.65%, *p* < 0.05) and RIPK3 KO (1.40 ± 0.37%, *p* < 0.01) groups (Fig. 4A). The selective expansion of Sox10^high^ populations suggests altered oligodendrocyte specification and maturation dynamics, consistent with Sox10’s established role as a key regulator of oligodendrocyte development [66]. We also observed a higher percentage of OligO4^high^ microglia (C25) in RIPK3 KO mice (0.20 ± 0.02%) compared to both WT (0.16 ± 0.03%, *p* < 0.01) and DKO (0.14 ± 0.02%, *p* < 0.001) groups. The co-expression of OligO4 with canonical microglial markers (CD11b and CD45) suggests these cells may represent a specialized population engaged in myelin phagocytosis, aligning with previously characterized myelin-engulfing microglia population [29, 67, 68].

Through secondary clustering, we identified distinct endothelial subpopulations with differential abundance across genotypes. Ly6C^high^ Endothelial Cells (C45) were significantly enriched in DKO mice (2.77 ± 0.42%) compared to WT (2.27 ± 0.37%, *p* < 0.05). Additionally, A2B5^high^Ncad^high^ Endothelial Cells (C56) showed significant elevation in both RIPK3 KO (0.72 ± 0.16%, *p* < 0.05) and DKO mice (0.91 ± 0.17%, *p* < 0.001) compared to WT controls (0.55 ± 0.12%), and further enrichment in DKO compared to RIPK3 KO mice (*p* < 0.05). We also observed pronounced differences in nonneural populations. DKO showed significant increase in both PDGFRa^high^p75NTR^high^ Nonneural Cells (C54) (1.29 ± 0.37% vs WT: 0.99 ± 0.15%, *p* < 0.05) and TrkB^high^p75NTR^low^Ncad^high^ Nonneural Cells (C55) (1.33 ± 0.42% vs WT: 0.81 ± 0.34%, *p* < 0.01). Additionally, p75NTR^low^Ncad^high^ Nonneural Cells (C51) showed a distinct pattern, with RIPK3 KO (0.87 ± 0.27%) showed significant lower abundance compared to both WT (1.38 ± 0.38%, *p* < 0.05) and DKO (1.56 ± 0.72%, *p* < 0.01). These populations likely represent vascular smooth muscle cells based on their marker profiles [69–71]. The concurrent expansion of these populations with endothelial cells suggests coordinated regulation of the neurovascular unit during development. We also noticed a significant decrease of PDGFRa^high^p75NTR^high^ALDH1A1^high^ Astrocytes (C48) in both RIPK3 KO (1.49 ± 0.29%) and DKO (1.58 ± 0.22%), compared with WT (2.13 ± 0.24%).

To validate these mass cytometry findings, we performed PECAM (CD31) immunostaining to assess vascular coverage. We found increased vascular coverage in the DKO cortex (1.97 ± 0.15%) compared to WT controls (1.47 ± 0.11%, *p* < 0.05), with RIPK3 KO mice displayed an intermediate phenotype (1.62 ± 0.13%) (Fig. 4C).

### Extrinsic apoptosis and necroptosis are differentially required to regulate select glia precursor numbers

**Fig. 5:**
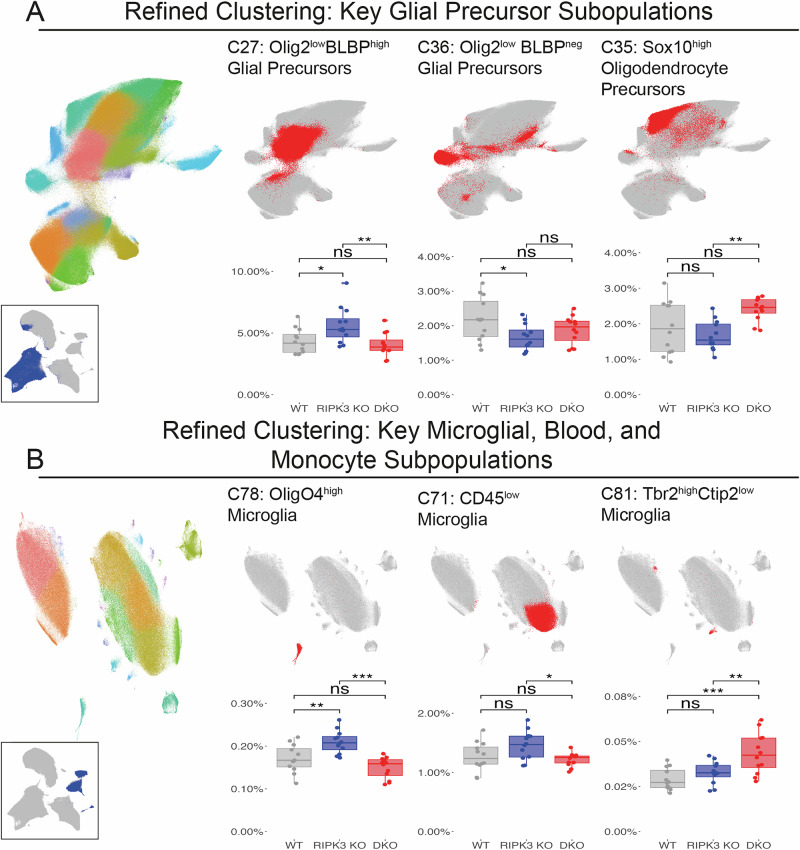
Subclustering analysis reveals differential glial subpopulation responses to necroptotic and extrinsic apoptotic pathway disruption. **A** Refined clustering analysis showing UMAP visualization and quantification of key glial lineage subpopulations. Left UMAP plot displays 22 distinct glial subclusters (C27–C44) identified through iterative Leiden clustering (colored overlay), with inset showing the analyzed clusters from initial clustering (dark blue). Representative UMAP plots and quantification are shown for three key glial subpopulations. **B** Refined clustering analysis showing UMAP visualization and quantification of key microglial, blood, and monocyte subpopulations. Left UMAP plot displays 23 distinct microglial, blood, and monocyte subclusters (C67–C89) identified through iterative Leiden clustering (colored overlay), with inset showing the analyzed clusters from initial clustering (dark blue). Representative UMAP plots and quantification are shown for three microglial, blood, and monocyte subpopulations. For each cluster, relative abundance% is compared across WT, RIPK3 KO, and DKO.

Detailed subclustering analysis of glial precursor populations revealed distinct effects of RIPK3 and Caspase-8 deletion on specific cellular subsets (Fig. 5A). Within the glial lineage, we identified three key precursor populations with differential responses to genetic perturbation: Olig2^low^BLBP^high^ Glial Precursors (C27), Sox10^high^ Oligodendrocyte Precursors (C35), and Olig2^low^BLBP^neg^ Glial Precursors (C36). Notably, RIPK3 deletion triggered opposing effects in BLBP-expressing versus BLBP-negative populations: C27 (BLBP^high^) cells expanded significantly in RIPK3 KO mice (5.63 ± 1.48% vs WT: 4.33 ± 1.00%, *p* < 0.05), while C36 (BLBP^neg^) cells showed a marked reduction (1.66 ± 0.38% vs WT: 2.18 ± 0.64%, *p* < 0.05). This divergent response suggests cell-type-specific roles for death receptor signaling in regulating distinct glial precursor populations.

### Differential effects of death receptor pathway disruption on Cargo Laden versus unladen microglia numbers

Reclustering of microglial, blood and monocyte subpopulations, we identified three key microglial subpopulations with differential responses to genetic perturbation: CD45^low^ Microglia (C71), OligO4^high^ Microglia (C78), and Tbr2^high^Ctip2^low^ Microglia (C81) (Fig. 5B). The OligO4^high^ subpopulation showed particular sensitivity to RIPK3 deletion, with significant expansion in RIPK3 KO mice (0.21 ± 0.03%) compared to both WT (0.17 ± 0.03%, *p* < 0.01) and DKO groups (0.15 ± 0.02%, *p* < 0.001). This finding suggests a specific role for RIPK3-dependent signaling in regulating this microglial subset, independent of Caspase-8 function.

## Discussion

Our single-cell mass cytometry analysis of the developing telencephalon reveals that extrinsic apoptosis and necroptosis operate in parallel with intrinsic apoptosis to regulate cell number during CNS development. In our study, the combined deletion of RIPK3 and Caspase-8 resulted in a 29% expansion of Tbr2⁺ intermediate progenitors and a 34% increase in vascular coverage, while cleaved Caspase-3 (CC3) levels remained largely unchanged. These quantitative changes challenge the traditional view that intrinsic apoptosis alone governs developmental cell death [4–10] and instead underscore the nonredundant, cell–type–specific functions of both death receptor-mediated pathways.

**Fig. 6:**
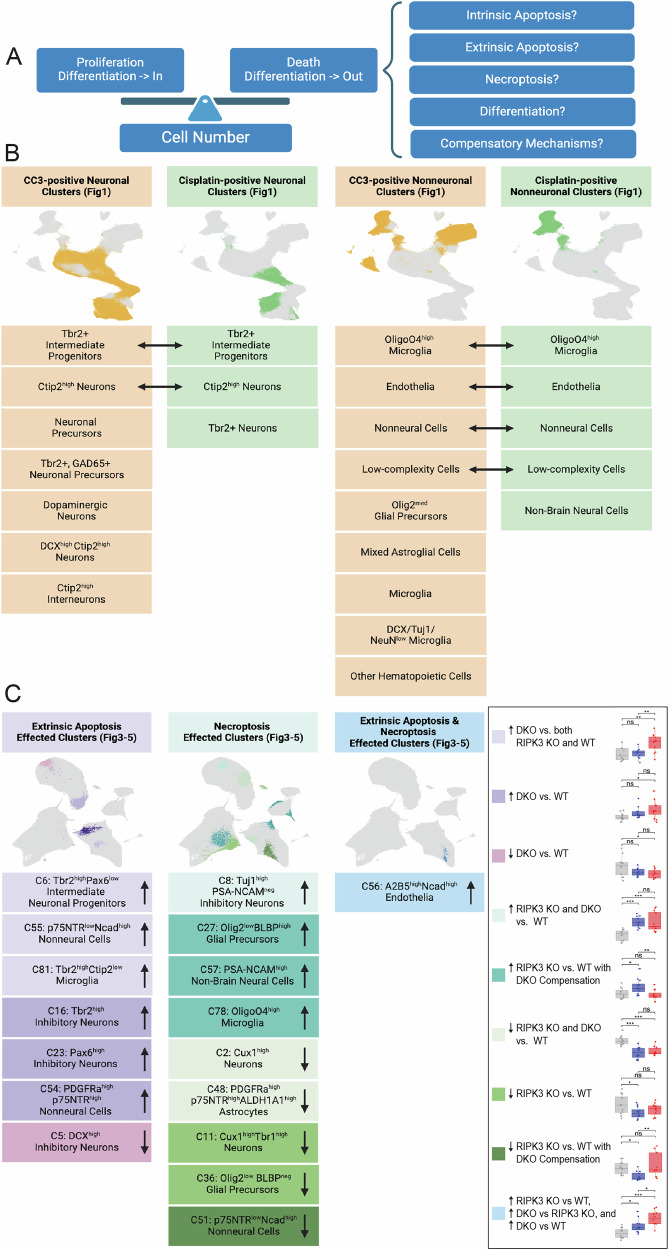
Comprehensive analysis of death pathway-specific effects on telencephalic cell population dynamics. **A** Model of developmental cell number regulation. Left: Cell numbers are balanced between input processes (proliferation and differentiation into the population) and output mechanisms (cell death and differentiation into other populations). Right: Potential mechanisms regulating developmental cell reduction, including distinct death pathways (apoptosis, necroptosis), lineage transitions, and compensatory responses. **B** Summary of cell populations with distinct death signatures in the developing telencephalon (Fig. 1, Supplementary Fig. S1, S2). UMAP visualization (top) and cluster identities (bottom) for neuronal (left) and nonneuronal (right) populations. Clusters showing cleaved Caspase-3 positivity are highlighted in orange, while those with Cisplatin uptake are shown in green. Bidirectional arrows indicate populations positive for both markers. **C** Summary of cell populations affected by death pathway deletion (Figs. 3–5). UMAP visualization (top) and cluster identities (bottom) showing populations regulated by: extrinsic apoptosis (left, purple, unique to DKO), necroptosis (middle, teal/green, RIPK3-dependent), or both pathways (right, blue, sequential changes across genotypes). Arrows indicate increased (↑) or decreased (↓) cluster abundance in knockout conditions.

Cell numbers in development reflect a dynamic equilibrium between proliferation, differentiation, and death (Fig. 6A). The observed changes in population percentages may arise through multiple mechanisms, including intrinsic apoptosis, extrinsic apoptosis, necroptosis, cell state transitions, and compensatory responses. The contribution of each death pathway varies by cell type, as evidenced by the population-specific responses to RIPK3 and Caspase-8 deletion we observed.

This cell type-specific regulation is clearly illustrated in our reanalysis of E13-P4 WT telencephalon (Fig. 6B), where we observed distinct death signatures across neuronal and non-neuronal populations. Several populations show evidence of utilizing multiple death pathways, as indicated by the bidirectional arrows between corresponding cell populations. These dual-positive populations include Tbr2^+^ intermediate progenitors and Ctip2^high^ neurons in the neuronal lineages, as well as OligO4^high^ microglia, endothelia, nonneural cells, and low-complexity cells in the non-neuronal populations. This overlap suggests these populations may employ multiple death mechanisms during development, potentially providing redundancy or context-specific regulation of cell numbers.

Notably, our genetic perturbation studies reveal three distinct patterns of pathway engagement. Some populations are uniquely affected in the RIPK3/Caspase-8 DKO but not in the RIPK3 single knockout, implicating a specific role for extrinsic apoptosis (e.g., Tbr2^high^Pax6^low^ intermediate progenitors). Other populations appear regulated primarily by RIPK3-dependent mechanisms (e.g., Tuj1^high^PSA-NCAM^neg^ inhibitory neurons), whereas certain cell types, such as A2B5^high^Ncad^high^ endothelial cells, require coordinated input from both pathways (Fig. 6C). This diversity in cell death regulation may reflect evolutionary adaptations that tailor death pathway engagement to the developmental requirements of specific cellular contexts [72].

A particularly striking aspect of our study is the selective expansion of the endothelial compartment in DKO mice. Our data suggest that telencephalic endothelial cells express high levels of Caspase-8 and RIPK3 mRNA (Fig. 2B). This expression pattern indicates that endothelial cells possess the molecular machinery for extrinsic apoptosis and necroptosis, making them particularly susceptible to death receptor signaling. When this signaling is blocked in DKO mice, ~34% more PECAM⁺ endothelial cells persist.

We interpret this ~34% increase as a consequence of blocking death‑receptor signaling, rather than evidence of a distinct angiogenic program. Two non‑exclusive mechanisms could explain the surplus. (1) Direct, cell‑autonomous survival: endothelial cells that would normally be eliminated by extrinsic apoptosis or necroptosis now persist. (2) Reciprocal neurovascular crosstalk: a transiently enlarged vascular bed delivers extra oxygen, nutrients, and angiocrine factors that sustain the ~29% expansion of the Tbr2⁺ intermediate progenitor pool; in turn, the larger progenitor population may secrete additional pro‑angiogenic cues, establishing a positive feedback loop. Whether this transient excess ultimately aids or hampers cerebrovascular maturation will depend on blood–brain‑barrier integrity, perfusion efficiency, and endothelial trophic output—parameters that remain to be measured in vivo. Moreover, coordinated pruning normally streamlines the embryonic vascular tree [73]; determining whether that remodeling is merely delayed or fundamentally disrupted in DKO brains is an important next step.

Beyond vascular changes, we also detected genotype-dependent shifts in membrane permeability: RIPK3 deletion alone cut Cisplatin uptake by >80% (*p* < 0.01), consistent with necroptosis contributing to membrane rupture during development. However, in DKO mice, where both necroptosis and Caspase-8–dependent extrinsic apoptosis are disabled, Cisplatin uptake was partially restored to ~61% of wild-type levels despite unchanged cleaved caspase-3 levels. This partial recovery in the absence of both death-receptor pathways suggests the presence of an alternative, compensatory mechanism.

We speculate that loss of both death-receptor arms unmasks a caspase-independent lytic pathway. A likely candidate is AIM2-inflammasome–driven pyroptotic death pathway: cytosolic DNA in the rapidly proliferating P4 telencephalon could activate AIM2, triggering Caspase-1/11-mediated GSDMD pore formation and permitting small molecules such as Cisplatin to enter without activating classical apoptotic markers [74–76]. Future work will test this model by quantifying inflammasome component expression and GSDMD-N fragments in vivo. Other lytic pathways, such as MPT-driven necrosis or PANoptosis, cannot be excluded, whereas ferroptosis seems unlikely given the iron-laden, lipid-peroxidation prerequisites absent in normal P4 cortex.

Notably, cell‑number changes remain largely confined to 20 of 89 refined subclusters (principally endothelial-related and progenitor subsets), arguing against widespread secondary activation of death pathways by extracellular damage‑associated molecules. However, we cannot formally exclude this possibility; metabolites released during abortive extrinsic apoptosis or necroptosis (e.g., ROS or nuclear DAMPs) may engage adjacent cell-death programs in neighboring cells via paracrine or feedforward mechanisms. This will be an interesting topic for future work.

Our analyses at a single developmental timepoint (P4) capture a critical window of postnatal pruning [42, 44] but do not resolve the temporal dynamics of pathway engagement. Future studies employing inducible Cre-loxP systems to delete RIPK3 and Caspase-8 at defined developmental stages are needed to delineate stage-specific requirements. In addition, conditional deletion in specific cell types (e.g., endothelial-specific deletion using Tie2-Cre or Cdh5-CreERT2) will help distinguish primary, cell-autonomous effects from secondary consequences on neural populations.

Finally, while our study focuses on extrinsic apoptosis and necroptosis, other regulated cell death mechanisms—such as pyroptosis and ferroptosis—may also contribute to developmental cell death [74, 77, 78]. Integration of spatial transcriptomics with proteomic profiling of death effector complexes could reveal how these pathways interact to maintain the delicate balance of cell number during brain development, with implications for understanding neurodevelopmental disorders associated with aberrant cell death [79].

In summary, our findings establish a revised framework in which multiple, context-dependent death pathways collaborate to refine brain architecture. By revealing nonredundant roles for extrinsic apoptosis and necroptosis in regulating specific neural and vascular populations, our study not only challenges longstanding paradigms but also provides new insights into the mechanistic underpinnings of CNS development.

## Materials and Methods

### Animals

All animal husbandry and experiments followed the Association for Assessment of Laboratory Animal Care guidelines. Mice were housed under controlled environmental conditions, including a 12 h light/dark cycle, ad libitum access to food and water, and maintained at 21 °C with 45–50% humidity. For experimental purposes, P4 pups were harvested from C57/BL6 wild-type, RIPK3 KO, and DKO mice bred in-house. Primers used for genotyping were Casp8 (5′-GGATGTCCAGGAAAAGATTTGTGTC-3′, 5′-CCTTCCTGAGTACTGTCACCTGT-3′) and RIPK3 (5′-CGCTTTAGAAGCCTTCAGGTTGAC-3′, 5′-GCAGGCTCTGGTGACAAGATTCATGG-3′, 5′-CCA-GAGGCCACTTGTGTAGCG-3′) (Integrated DNA Technologies).

Pup sexing was based on differences in anogenital pigmentation: male pups exhibit a pigmented spot that eventually develops into the scrotum, while female pups lack this marker [80].

### Brain tissue processing and single-cell dissociation

P4 WT, RIPK3 KO, and DKO mice were decapitated, and their brains were dissected into four regions: telencephalon, diencephalon, mesencephalon, and rhombencephalon. Single-cell separation was achieved using a modified version of Volovitz and colleagues’ method [81]. Brain tissue was enzymatically digested in a solution containing dispase II (Sigma-Aldrich, D4693), collagenase type IV (Worthington, LS004186), DNAse-I (Sigma-Aldrich, 11284932001), and hyaluronidase (Sigma-Aldrich, H3884). After mechanical dissociation using a P1000 pipette and 30 min incubation at 37 °C, the cell suspension was filtered through a 45 μm mesh, centrifuged at 300 x g for 5 min at 4 °C, and resuspended in DPBS (DPBS; Thermo Fisher Scientific, 14190) for subsequent Cisplatin staining.

### Cisplatin staining, fixation and cell counting

Cells were incubated with 10 µM Cisplatin (Sigma Aldrich, P4394) for 30 s. The reaction was quenched and diluted 1:10 with DPBS containing 0.5% bovine serum albumin (BSA; Sigma-Aldrich, A9418) and immediately centrifuged at 300 x g for 3 min at 4 °C. After centrifugation and resuspension, cells were fixed with 1.6% paraformaldehyde (PFA; Electron Microscopy Services, CAS 30525-89-4) for 10 min. Following a DPBS wash, cells were prepared in cell staining medium (CSM; 0.5% BSA, 0.02% NaN_3_ in PBS). A 100 μL aliquot was reserved for flow cytometry quality control, and the remaining sample was stored at −80 °C. Dissociation quality was first confirmed by microscopic inspection using an EVOS AMF4300 microscope and later through flow cytometry. Samples with predominantly single cells and minimal debris were counted using a Bio-Rad TC20 Automated Cell Counter.

### Sample barcoding, staining, and intercalation for mass cytometry

Telencephalon samples from P4 RIPK3 KO mice, DKO mice, and WT mice were processed for mass cytometry using an established protocol [29]. Samples were barcoded, pooled for antibody staining, and intercalated. The surface and intracellular antibodies are listed in Supplementary Table 1. The analysis was conducted on a CYTOF XT System (Fluidigm Corporation) by the University of Virginia Flow Cytometry Core.

### Normalization, debarcoding, gating, and compensation

Raw FCS data files were standardized through bead normalization and debarcoded [82, 83]. Single-cell isolation was performed via cleanup gating using Cytobank (Supplementary Fig. S8C–E). To minimize signal spillover between channels, a manual compensation matrix was applied [84], adapted from Chevrier et al. The resulting high-quality, single-cell data for each sample were exported as individual.fcs files for subsequent analysis.

### Batch correction

To mitigate batch effects and ensure consistent antibody signals across barcode sets, batch correction was performed [85]. A universal sample containing cells from all three genotypes was used to standardize signals. Antibodies producing Gaussian distributions with mean signal variance >1% were corrected at the 50th percentile (e.g., PDGFRa). CD133, Ki67, and GFAP were corrected at the 95th percentile. Other markers with low variance ( < 1%) were not batch corrected.

### Customized Cisplatin gate

To keep live/dead discrimination consistent despite technical variation in ¹⁹⁵Pt-Cisplatin staining, we applied a *proportional* gating rule: the live-cell gate is placed 1.75 × above the background intensity. In 21 of 22 telencephalic samples, the background mode was ~200 counts, yielding the standard gate at 350 counts. One E14 sample showed a uniformly elevated baseline ( ~ 1100 counts; Supplementary Fig. S3A, red histogram). Applying the same 1.75 × factor positioned the gate at 1925 counts (Supplementary Fig. S3B). This adjustment preserves the relative separation between viable and non-viable events while accommodating baseline drift. The outlier is removed from the main trend analysis (Fig. 1) to prevent axis compression but is included—re-gated—in Supplementary Fig. S3.

### Clustering of high-dimensional data

Cells were clustered using 37 expression markers, with populations identified based on established CNS marker profiles [29]. The data were dimensionally reduced using uniform manifold approximation and projection (UMAP) for visualization [51, 86], with parameters: nearest neighbors = 15, metric = Euclidean, local connectivity = 1, components = 2, epochs = 1000.

### Tissue preparation and immunohistochemistry

P4 mouse brains were fixed (4% PFA, 48 h), dehydrated (30% sucrose, 72 h), frozen (−80 °C), embedded in OCT, and cryosectioned (30 μm).

Mounted sections were washed with PBS, blocked (2% normal donkey serum, 1%BSA, 0.1% Triton, 0.05% Tween-20 in PBS, 1 h), and incubated with primary antibodies overnight at 4 °C: PECAM (PE anti-mouse CD31, rat, 1:300, BioLegend, catalog 160203), NeuN (NeuN, rabbit, 1:1000, Abcam, catalog ab177487) and Tbr2 (EOMES, rat, 1:300, Invitrogen, catalog 14-4875-82). After PBST (PBS + 0.05% Tween 20) washes, sections were incubated with Alexa Fluor secondary antibodies (1:500; Thermo Fisher Scientific; 2 h), washed with PBS, and mounted with Fluoromount-G (SouthernBiotech, catalog 0100-01).

### RNAscope

Brain sections on charged slides were air-dried (36 h), treated with H_2_O_2_ (RNAscope H_2_O_2_ and Protease Reagents Kit, Advanced Cell Diagnostics, catalog 322381, 10 min), and incubated with protease IV (RNAscope H2O2 and Protease Reagents Kit, Advanced Cell Diagnostics, catalog 322381, 15 min, 40 °C). After washing, the tissue was incubated in Probe Master Mix for 2 h at 40 °C (Probe1: Mm-RipK3, catalog 462541; Probe2: Mm-Casp8-C2, catalog 468971-C2; Advanced Cell Diagnostics). Samples were then amplified using AMP1-3 and visualized with TSA Cyanine 5 and TSA Cyanine 3 staining.

### Image analysis

Images were taken using a Zeiss 980 confocal laser-scanning microscope at ×20/0.8 magnification. All confocal microscopy images were processed using the “Fiji is Just ImageJ” application and set to maximum intensity projection.

For RNAscope quantification, images were separated into individual fluorescence channels (Casp8: cyan; RIPK3: magenta). Each channel was manually thresholded using *Image > Adjust > Threshold* to generate binary masks. Background noise was minimized using *Process > Binary > Open*, and puncta were expanded with six iterations of *Process > Binary > Dilate*, corresponding to an approximate 6-pixel outward expansion in all directions.

Puncta in each channel were quantified using *Analyze > Analyze Particles*, with a minimum size threshold applied to exclude small background artifacts. Colocalization was assessed by performing an *AND* operation between the two binary masks (*Process > Image Calculator*), and the resulting overlap image was analyzed for colocalized puncta.

Colocalization was calculated as:

- $$\%\, {{{\rm{magenta}}}}\; {{{\rm{colocalizing}}}}\; {{{\rm{with}}}}\; {{{\rm{cyan}}}}=({{{\rm{overlapping}}}}\; {{{\rm{puncta}}}}/{{{\rm{total}}}}\; {{{\rm{magenta}}}}\; {{{\rm{puncta}}}})\times 100$$

For Tbr2^+^ cell number quantification, images were separated into individual channels, and the Tbr2 channel was isolated for further analysis. Brightness and contrast adjustments were made to maximize positive signal intensity and minimize background intensity. The entire image was then analyzed using the “QuPath” application. The “Polygon” tool was used to outline the region of interest (ROI) and the “Cell detection” tool to identify Tbr2^+^ cells. Manual checks were performed to ensure that all positive cells were detected accurately. In total,2 images from each section with 3 sections per animal were included in the analysis.

For PECAM^+^ area quantification, images were again separated into individual channels, and the PECAM channel was isolated for further analysis. Image brightness and contrast were adjusted to optimize signal detection. The Labkit Pixel Classification plugin was used to classify the entire image, with representative foreground and background pen markings adjusted manually to improve positive signal detection accuracy. The segmentation result was reimported to ImageJ and thresholded to separate “foreground” and “background” pixel classes. Each section image was analyzed independently. The Analyze > Measure function was used to quantify the %Area positive for PECAM. In total, 3 images from each section with 3 sections per animal were included in the analysis.

### Statistical analysis

Sample-size determination: sample sizes for comparisons among WT, RIPK3, and DKO mice were guided by prior experience in our laboratory. We aimed for a minimum of 8 animals per group whenever possible. Although no formal power analysis was performed for each experiment, previous calculations using Cohen’s *d* (α = 0.05, power = 0.80) show that ~6 animals per group can detect a ≈ 40% difference in means given a 25% standard deviation, whereas ~12 animals are needed to detect a ≈ 20% difference with a 20% standard deviation. Accordingly, the comparison study in this work used 6–12 animals per group.

Randomization: no formal randomization was applied because group identity was determined solely by genotype; pups of different genotypes were processed together during sample barcoding and staining in mixed batches to minimize technical bias.

Blinding and data handling: slides and flow-cytometry files were coded by an independent colleague, and investigators performing imaging, gating, and statistical analyses were blinded to genotype until all primary quantification was complete.

Modelling developmental trajectories: for time course experiments, developmental age was treated as a continuous, ordered variable to reflect its inherent temporal structure. To assess overall trends in marker-positive cell percentages (e.g., CC3^+^, Cisplatin^+^, Ki67^+^), we applied linear regression models, using developmental age as the sole predictor. The statistical significance of temporal changes was determined by testing whether the regression slope differed from zero, with results reported as *p*-values and R² to quantify model fit.

To visualize nonlinear developmental trajectories, we used locally estimated scatterplot smoothing (LOESS) with 95% confidence intervals. This nonparametric approach provides a flexible fit that captures gradual changes over time without assuming a specific functional form.

Group comparisons: statistical comparisons among WT, RIPK3, and DKO were primarily performed using one-way ANOVA followed by Tukey’s post-hoc test for multiple comparisons, after verifying normality (Q-Q plot) and equal variances (Levene).

For cell count analyses involving genotype and sex factors, two-way ANOVA was performed to assess main effects and interactions. Post-hoc pairwise comparisons were conducted using estimated marginal means (emmeans) with Tukey’s adjustment for multiple comparisons.

For direct comparisons between two groups in heatmap, Student’s t-test was applied. Statistical significance levels were defined as: *p < 0.05, **p < 0.01, ***p < 0.001, with non-significant differences denoted as “ns”.

## Supplementary information

Supplementary materials

## Data Availability

All raw mass cytometry data generated in this study are publicly accessible through the Flow Repository (ID: FR-FCM-Z934, https://flowrepository.org/). The corresponding FCS files for Fig. 1 (single-cell mass cytometry of mouse telencephalon from E13 to P4, including live and dying cells), along with the gating strategies used for preprocessing, are deposited in Cytobank (https://cytobank.org/cytobank/experiments/) under experiment IDs 105280-105284. The corresponding FCS files for P4 WT, RIPK3 KO, and DKO samples are available in Cytobank under experiment ID 123863.

## Code availability

All R (4.4.1) and Python (3.8) scripts used to preprocess*.fcs* files, run dimensionality reduction, and Leiden clustering are openly available at https://github.com/zunderlab/VanDeusen-et-al.-CNS-Development-Manuscript.
