# Supplementary material for: Extrinsic apoptosis and necroptosis in telencephalic development: a single-cell mass cytometry study
Source: Cell Death Differ. 2025 Oct 21;33(3):656–71. doi: 10.1038/s41418-025-01594-5 (PMC13035822; doi:10.1038/s41418-025-01594-5)
Supplement: Supplementary file 1 — Supplementary materials [file 41418_2025_1594_MOESM1_ESM.pdf]

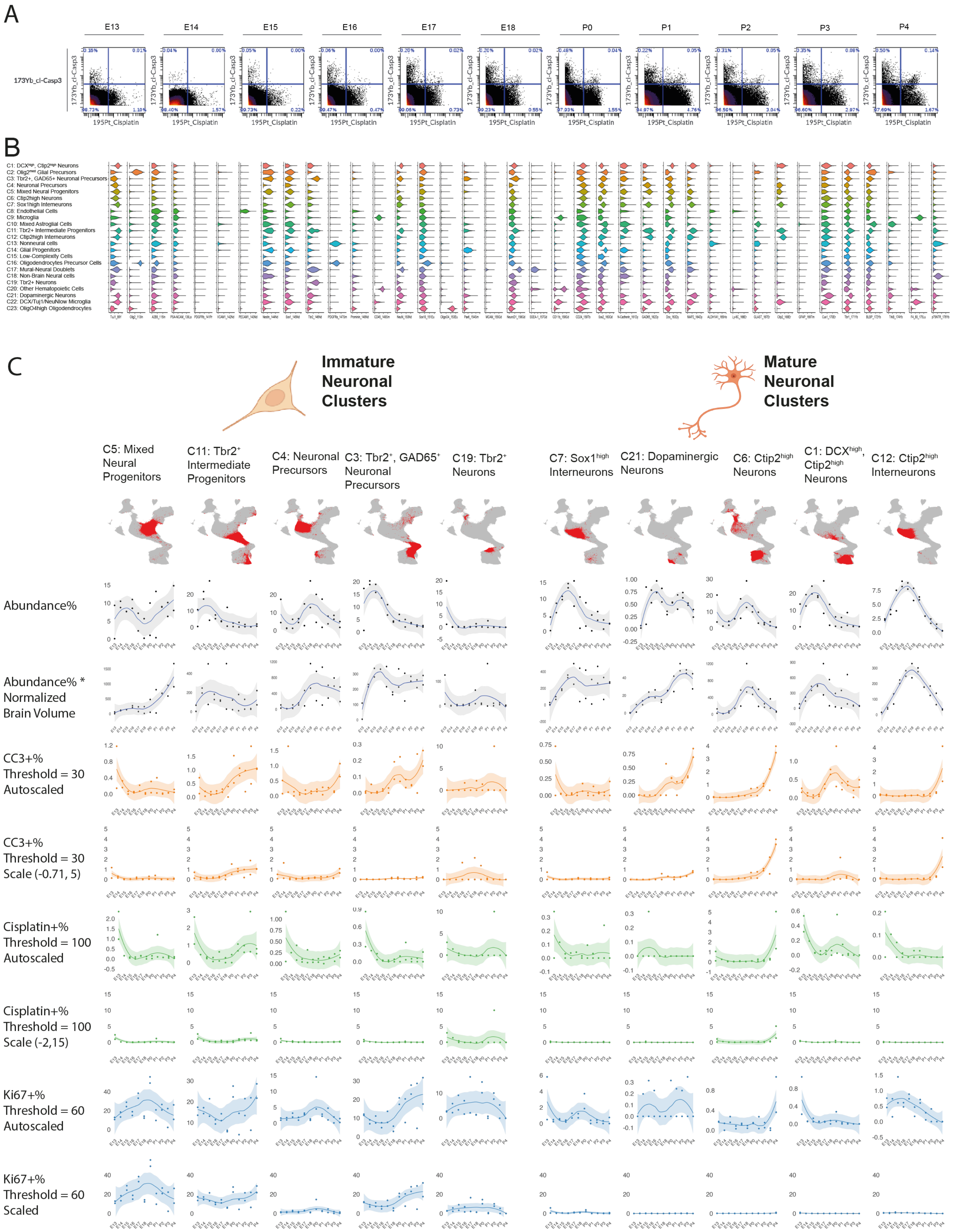
A
E13
E14
E15
E16
E17
E18
P0
P1
P2
P3
P4
173Yb_cl-Casp3
195Pt_Cisplatin
B
C1: DCXhigh, Ctip2high Neurons
C2: Olig2med Glial Precursors
C3: Tbr2+, GAD65+ Neuronal Precursors
C4: Neuronal Precursors
C5: Mixed Neural Progenitors
C6: Ctip2high Neurons
C7: Sox1high Interneurons
C8: Endothelial Cells
C9: Microglia
C10: Mixed Astroglial Cells
C11: Tbr2+ Intermediate Progenitors
C12: Ctip2high Interneurons
C13: Nonneural cells
C14: Glial Progenitors
C15: Low-Complexity Cells
C16: Oligodendrocytes Precursor Cells
C17: Mural-Neural Doublets
C18: Non-Brain Neural cells
C19: Tbr2+ Neurons
C20: Other Hematopoietic Cells
C21: Dopaminergic Neurons
C22: DCX/Tuj1/NeuNlow Microglia
C23: OligO4high Oligodendrocytes
C
Immature Neuronal Clusters
Mature Neuronal Clusters
C5: Mixed Neural Progenitors
C11: Tbr2+ Intermediate Progenitors
C4: Neuronal Precursors
C3: Tbr2+, GAD65+ Neuronal Precursors
C19: Tbr2+ Neurons
C7: Sox1high Interneurons
C21: Dopaminergic Neurons
C6: Ctip2high Neurons
C1: DCXhigh, Ctip2high Neurons
C12: Ctip2high Interneurons
Abundance%
Abundance% * Normalized Brain Volume
CC3+% Threshold = 30 Autoscaled
CC3+% Threshold = 30 Scale (-0.71, 5)
Cisplatin+% Threshold = 100 Autoscaled
Cisplatin+% Threshold = 100 Scale (-2,15)
Ki67+% Threshold = 60 Autoscaled
Ki67+% Threshold = 60 Scaled

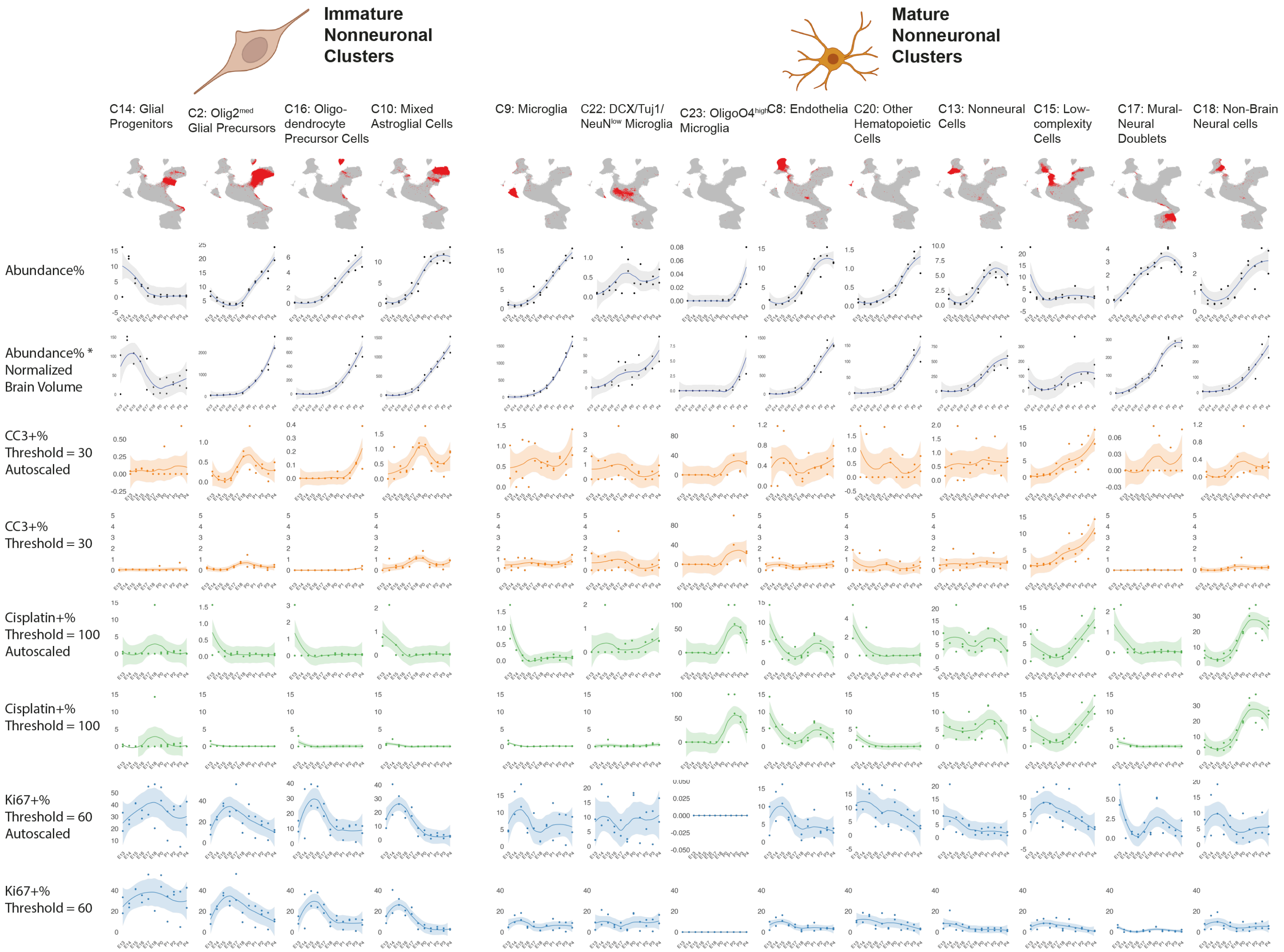

Immature Nonneuronal Clusters
Mature Nonneuronal Clusters
C14: Glial Progenitors
C2: Olig2med Glial Precursors
C16: Oligo-dendrocyte Precursor Cells
C10: Mixed Astroglial Cells
C9: Microglia
C22: DCX/Tuj1/ NeuNlow Microglia
C23: OligoO4high Microglia
C8: Endothelia
C20: Other Hematopoietic Cells
C13: Nonneural Cells
C15: Low-complexity Cells
C17: Mural-Neural Doublets
C18: Non-Brain Neural cells
Abundance%
Abundance% * Normalized Brain Volume
CC3+% Threshold = 30 Autoscaled
CC3+% Threshold = 30
Cisplatin+% Threshold = 100 Autoscaled
Cisplatin+% Threshold = 100
Ki67+% Threshold = 60 Autoscaled
Ki67+% Threshold = 60

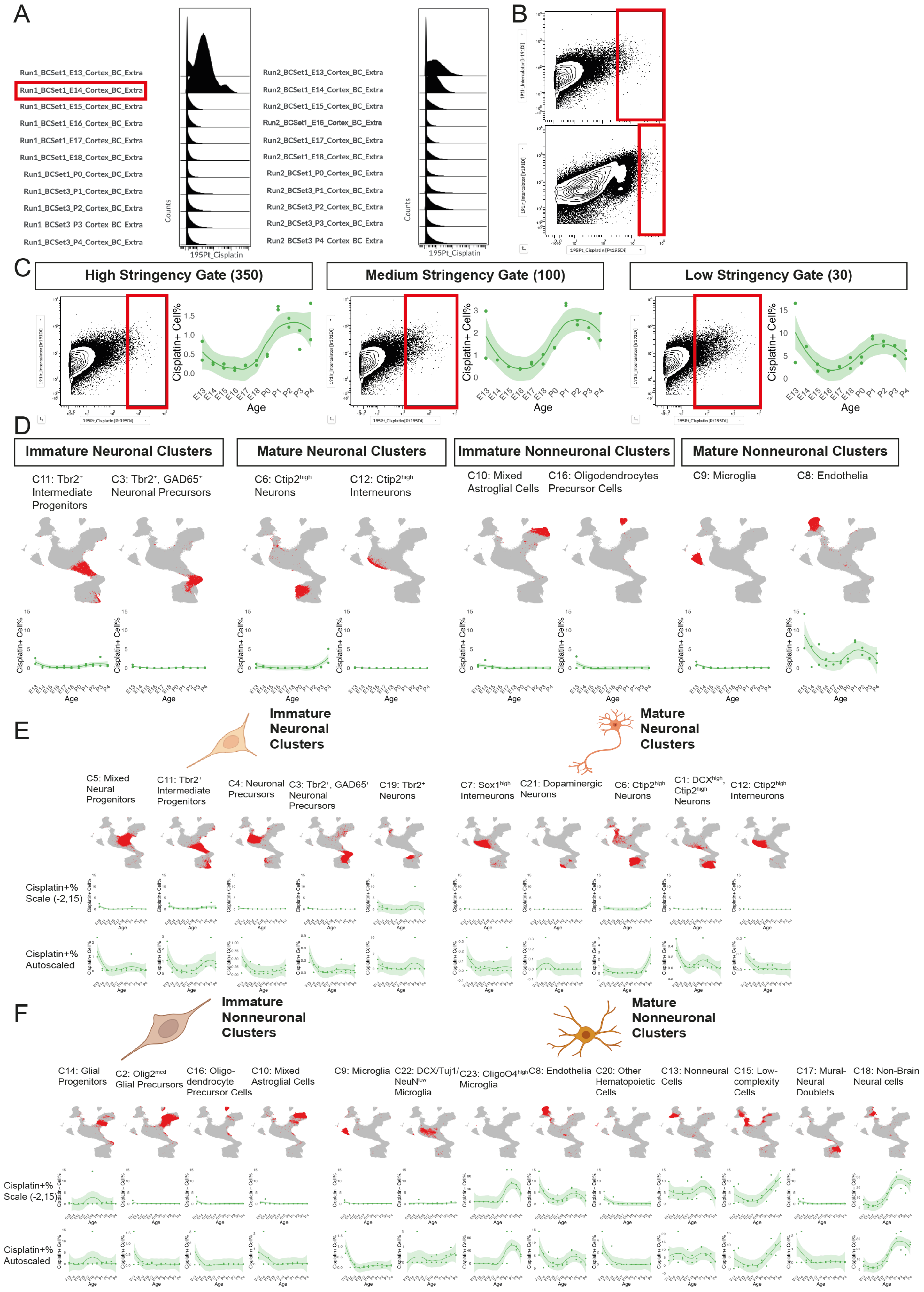

A
Run1_BCSet1_E13_Cortex_BC_Extra
Run1_BCSet1_E14_Cortex_BC_Extra
Run1_BCSet1_E15_Cortex_BC_Extra
Run1_BCSet1_E16_Cortex_BC_Extra
Run1_BCSet1_E17_Cortex_BC_Extra
Run1_BCSet1_E18_Cortex_BC_Extra
Run1_BCSet1_P0_Cortex_BC_Extra
Run1_BCSet3_P1_Cortex_BC_Extra
Run1_BCSet3_P2_Cortex_BC_Extra
Run1_BCSet3_P3_Cortex_BC_Extra
Run1_BCSet3_P4_Cortex_BC_Extra
Counts
195Pt_Cisplatin
Run2_BCSet1_E13_Cortex_BC_Extra
Run2_BCSet1_E14_Cortex_BC_Extra
Run2_BCSet1_E15_Cortex_BC_Extra
Run2_BCSet1_E16_Cortex_BC_Extra
Run2_BCSet1_E17_Cortex_BC_Extra
Run2_BCSet1_E18_Cortex_BC_Extra
Run2_BCSet1_P0_Cortex_BC_Extra
Run2_BCSet3_P1_Cortex_BC_Extra
Run2_BCSet3_P2_Cortex_BC_Extra
Run2_BCSet3_P3_Cortex_BC_Extra
Run2_BCSet3_P4_Cortex_BC_Extra
Counts
195Pt_Cisplatin
B
C
High Stringency Gate (350)
Medium Stringency Gate (100)
Low Stringency Gate (30)
Cisplatin+ Cell%
Age
E13 E14 E15 E16 E17 E18 P0 P1 P2 P3 P4
D
Immature Neuronal Clusters
Mature Neuronal Clusters
Immature Nonneuronal Clusters
Mature Nonneuronal Clusters
C11: Tbr2+ Intermediate Progenitors
C3: Tbr2+, GAD65+ Neuronal Precursors
C6: Ctip2high Neurons
C12: Ctip2high Interneurons
C10: Mixed Astroglial Cells
C16: Oligodendrocytes Precursor Cells
C9: Microglia
C8: Endothelia
Cisplatin+ Cell%
Age
E
Immature Neuronal Clusters
Mature Neuronal Clusters
C5: Mixed Neural Progenitors
C11: Tbr2+ Intermediate Progenitors
C4: Neuronal Precursors
C3: Tbr2+, GAD65+ Neuronal Precursors
C19: Tbr2+ Neurons
C7: Sox1high Interneurons
C21: Dopaminergic Neurons
C6: Ctip2high Neurons
C1: DCXhigh, Ctip2high Neurons
C12: Ctip2high Interneurons
Cisplatin+% Scale (-2,15)
Cisplatin+% Autoscaled
F
Immature Nonneuronal Clusters
Mature Nonneuronal Clusters
C14: Glial Progenitors
C2: Olig2med Glial Precursors
C16: Oligo-dendrocyte Precursor Cells
C10: Mixed Astroglial Cells
C9: Microglia
C22: DCX/Tuj1/ NeuNlow Microglia
C23: OligoO4high Microglia
C8: Endothelia
C20: Other Hematopoietic Cells
C13: Nonneural Cells
C15: Low-complexity Cells
C17: Mural-Neural Doublets
C18: Non-Brain Neural cells
Cisplatin+% Scale (-2,15)
Cisplatin+% Autoscaled

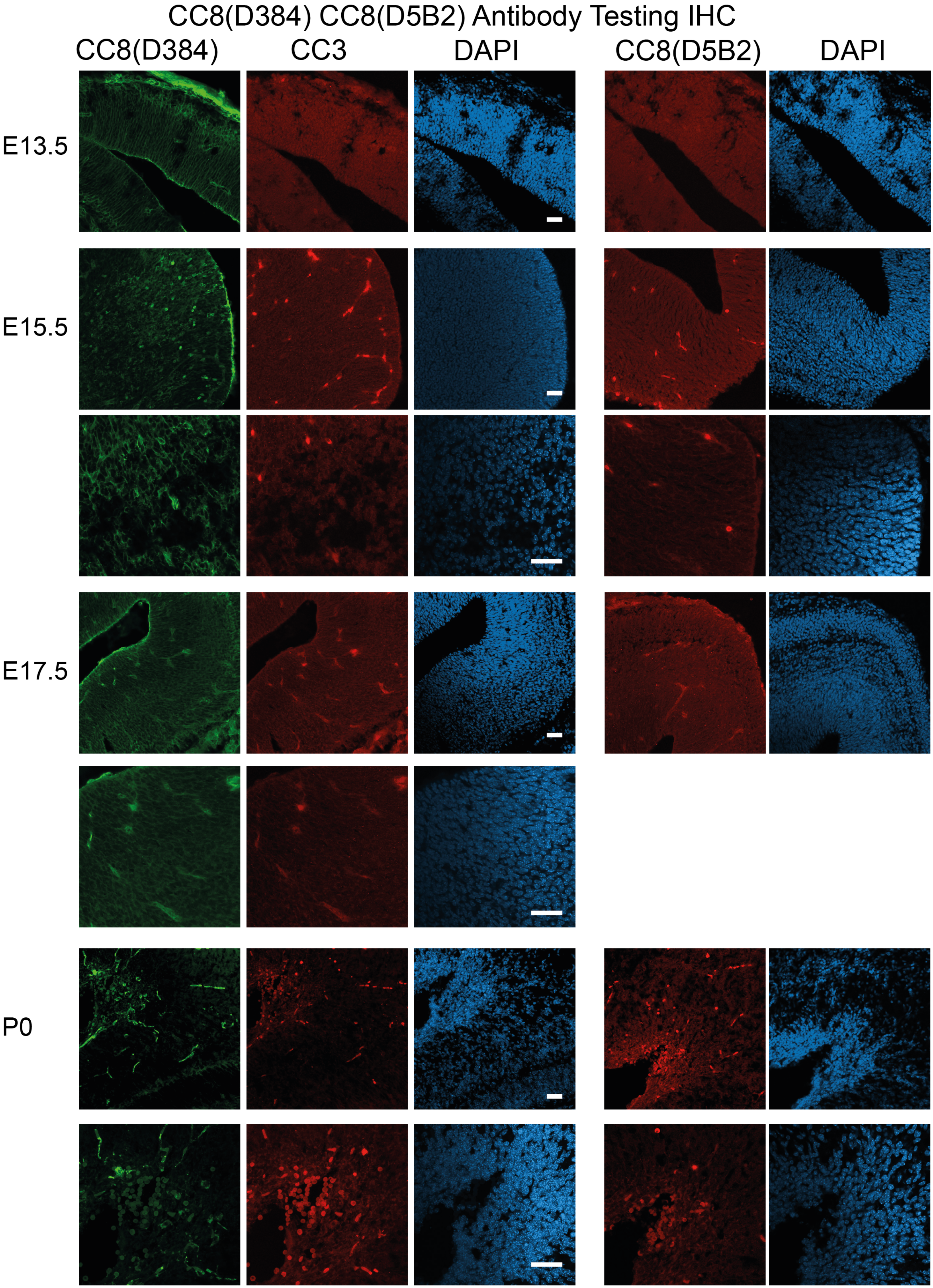
CC8(D384) CC8(D5B2) Antibody Testing IHC
CC8(D384)
CC3
DAPI
CC8(D5B2)
DAPI
E13.5
E15.5
E17.5
P0

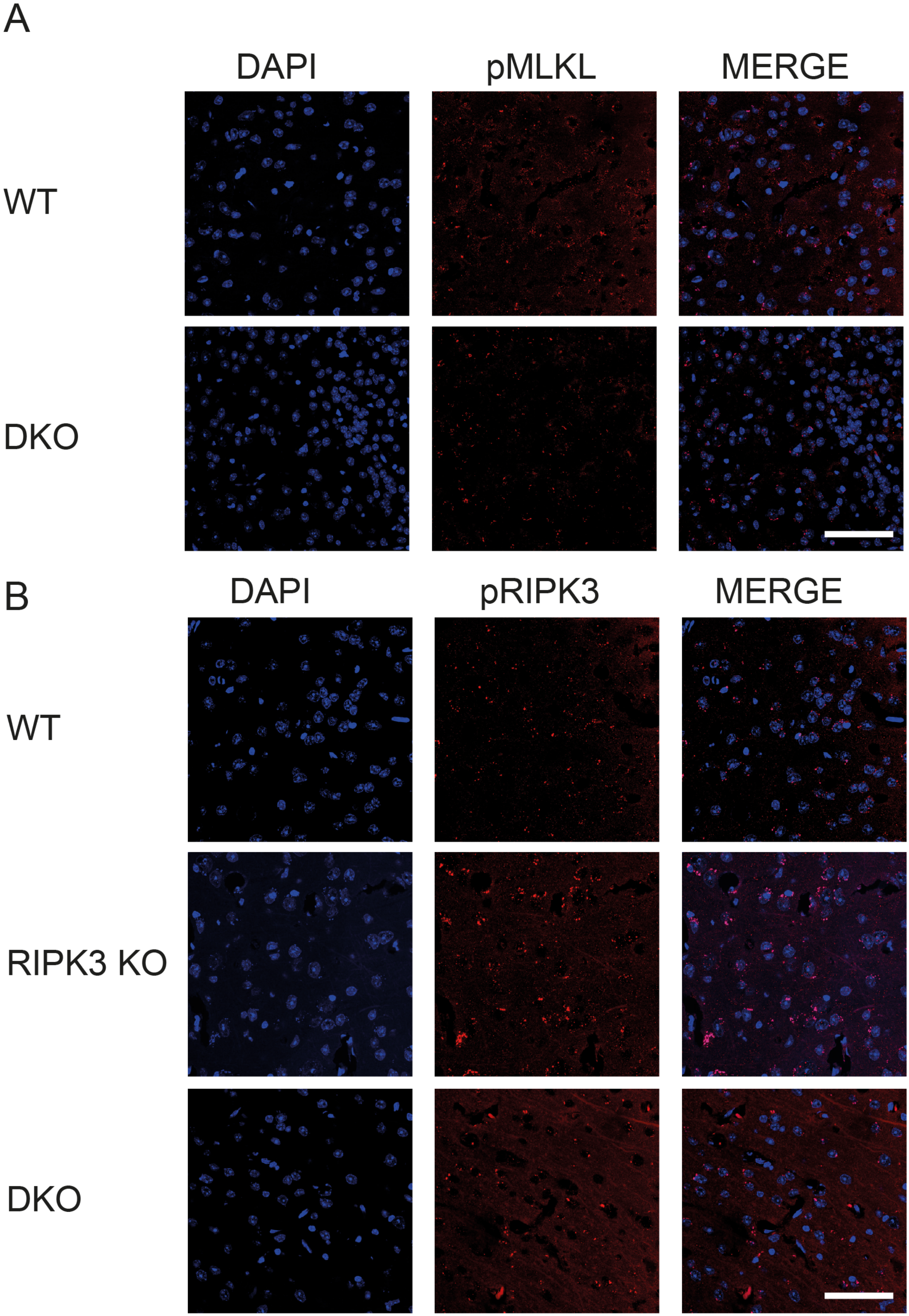
A
DAPI
pMLKL
MERGE
WT
DKO
B
DAPI
pRIPK3
MERGE
WT
RIPK3 KO
DKO

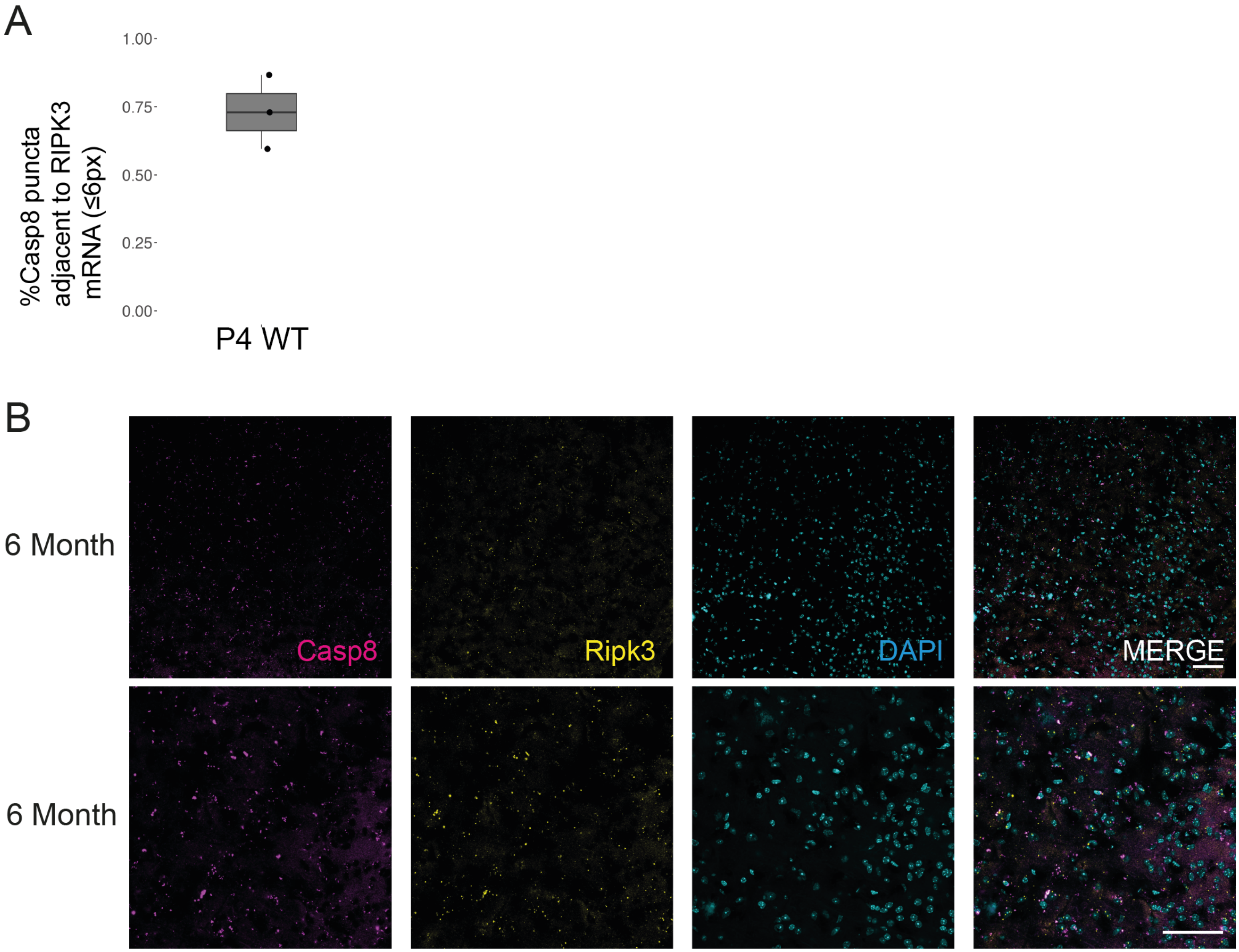
A
1.00
0.75
0.50
0.25
0.00
%Casp8 puncta adjacent to RIPK3 mRNA (≤6px)
P4 WT
B
6 Month
6 Month
Casp8
Ripk3
DAPI
MERGE

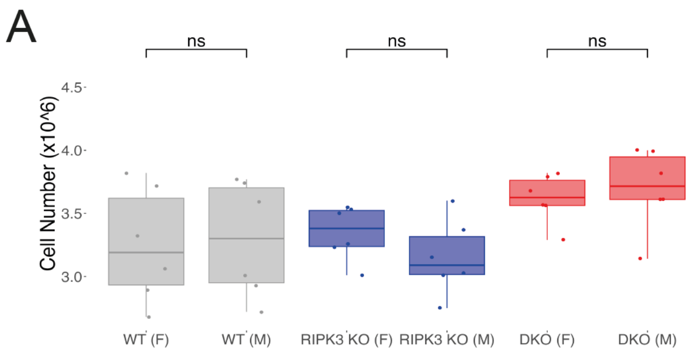
A
ns
ns
ns
Cell Number (x10^6)
4.5
4.0
3.5
3.0
WT (F)
WT (M)
RIPK3 KO (F)
RIPK3 KO (M)
DKO (F)
DKO (M)

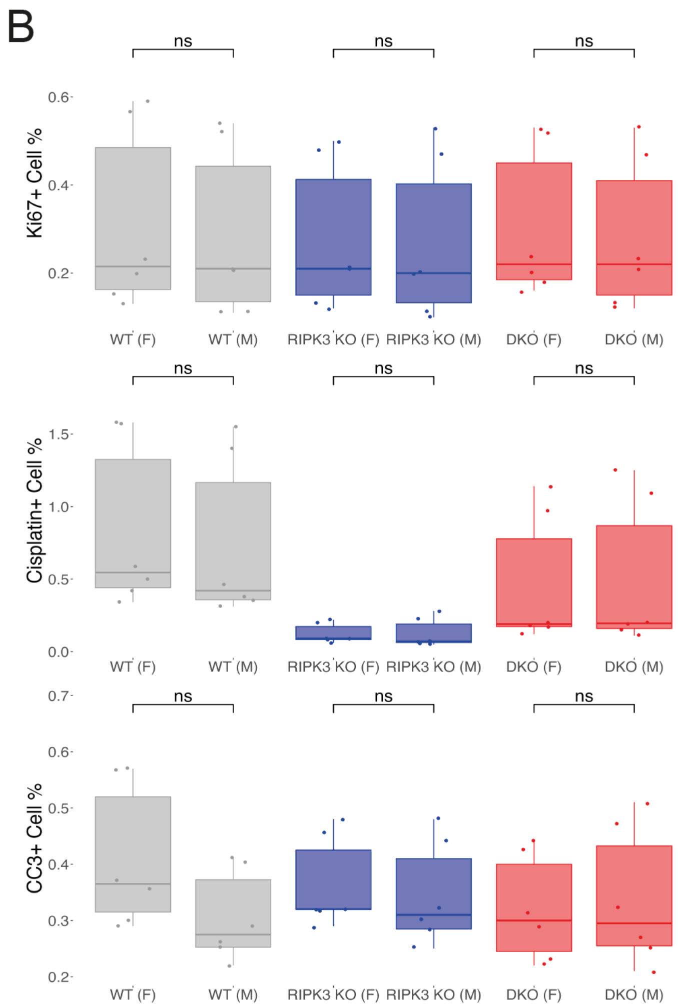
B
ns
ns
ns
Ki67+ Cell %
0.6
0.4
0.2
Cisplatin+ Cell %
1.5
1.0
0.5
0.0
CC3+ Cell %
0.7
0.6
0.5
0.4
0.3
0.2
WT (F)
WT (M)
RIPK3 KO (F)
RIPK3 KO (M)
DKO (F)
DKO (M)

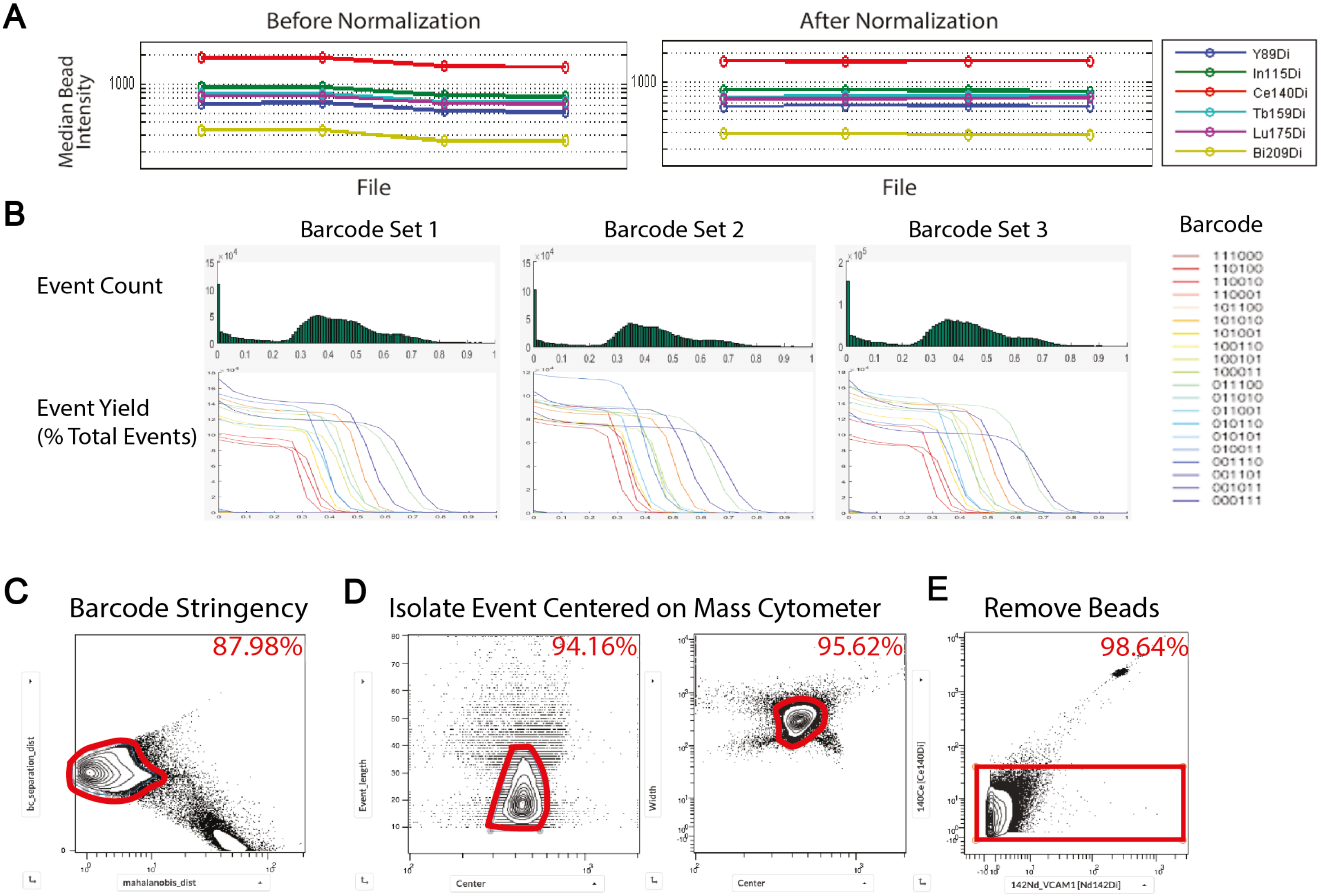


F

| | Pr141Di | Nd142Di | Nd143Di | Nd144Di | Nd145Di | Nd146Di | Sm147Di | Nd148Di | Sm149Di | Nd150Di | Eu151Di | Sm152Di | Eu153Di | Sm154Di | Gd155Di | Gd156Di | Gd157Di | Gd158Di | Tb159Di | Gd160Di | Dy161Di | Dy162Di | Dy163Di | Dy164Di | Ho165Di | Er166Di | Er167Di | Er168Di | Tm169Di | Er170Di | Yb171Di | Yb172Di | Yb173Di | Yb174Di | Lu175Di | Yb176Di |
|---|---|---|---|---|---|---|---|---|---|---|---|---|---|---|---|---|---|---|---|---|---|---|---|---|---|---|---|---|---|---|---|---|---|---|---|---|
| Pr141Di | 100 | 0 | 0 | 0 | 0 | 0 | 0 | 0 | 0 | 0 | 0 | 0 | 0 | 0 | 0 | 0 | 0 | 3 | 0 | 0 | 0 | 0 | 0 | 0 | 0 | 0 | 0 | 0 | 0 | 0 | 0 | 0 | 0 | 0 | 0 | 0 |
| Nd142Di | 0 | 100 | 0 | 0 | 0 | 0 | 0 | 0 | 0 | 0 | 0 | 0 | 0 | 0 | 0 | 0 | 0 | 3 | 0 | 0 | 0 | 0 | 0 | 0 | 0 | 0 | 0 | 0 | 0 | 0 | 0 | 0 | 0 | 0 | 0 | 0 |
| Nd143Di | 0 | 0 | 100 | 0 | 0 | 0 | 0 | 0 | 0 | 0 | 0 | 0 | 0 | 0 | 0 | 0 | 0 | 0 | 0 | 0 | 0 | 0 | 0 | 0 | 0 | 0 | 0 | 0 | 0 | 0 | 0 | 0 | 0 | 0 | 0 | 0 |
| Nd144Di | 0 | 0 | 0 | 100 | 0 | 0 | 0 | 0 | 0 | 0 | 0 | 0 | 0 | 0 | 0 | 0 | 0 | 0 | 0 | 0 | 0 | 0 | 0 | 0 | 0 | 0 | 0 | 0 | 0 | 0 | 0 | 0 | 0 | 0 | 0 | 0 |
| Nd145Di | 0 | 0 | 0.2 | 1 | 100 | 4 | 0 | 0.2 | 0 | 0 | 0 | 0 | 0 | 0 | 0 | 0 | 0 | 0 | 0 | 0 | 3 | 0 | 0 | 0 | 0 | 0 | 0 | 0 | 0 | 0 | 0 | 0 | 0 | 0 | 0 | 0 |
| Nd146Di | 0 | 0 | 0.03 | 0 | 0.3 | 100 | 0 | 0 | 0 | 0 | 0 | 0 | 0 | 0 | 0 | 0 | 0 | 0 | 0 | 0 | 0 | 3 | 0 | 0 | 0 | 0 | 0 | 0 | 0 | 0 | 0 | 0 | 0 | 0 | 0 | 0 |
| Sm147Di | 0 | 0 | 0.03 | 0.05 | 0.08 | 0.2 | 100 | 2.7 | 0.7 | 0.3 | 0.03 | 0.4 | 0.05 | 0.3 | 0 | 0 | 0 | 0 | 0 | 0 | 0 | 0 | 0.9 | 0 | 0 | 0 | 0 | 0 | 0 | 0 | 0 | 0 | 0 | 0 | 0 | 0 |
| Nd148Di | 0 | 0 | 0.15 | 0 | 0.2 | 0.8 | 0 | 100 | 0.45 | 0.3 | 0 | 0 | 0 | 0 | 0 | 0 | 0 | 0 | 0 | 0 | 0 | 0 | 0 | 3.5 | 0 | 0 | 0 | 0 | 0 | 0 | 0 | 0 | 0 | 0 | 0 | 0 |
| Sm149Di | 0 | 0 | 0 | 0 | 0 | 0.05 | 0 | 0.7 | 100 | 1.8 | 0.07 | 0.6 | 0.05 | 0.3 | 0 | 0 | 0 | 0 | 0 | 0 | 0 | 0 | 0 | 0 | 0.65 | 0 | 0 | 0 | 0 | 0 | 0 | 0 | 0 | 0 | 0 | 0 |
| Nd150Di | 0 | 0 | 0 | 0 | 0 | 0 | 0 | 0 | 0 | 100 | 0 | 0 | 0 | 0 | 0 | 0 | 0 | 0 | 0 | 0 | 0 | 0 | 0 | 0 | 0 | 0 | 0 | 0 | 0 | 0 | 0 | 0 | 0 | 0 | 0 | 0 |
| Eu151Di | 0 | 0 | 0 | 0 | 0 | 0 | 0 | 0 | 0 | 0 | 100 | 0 | 0 | 0 | 0 | 0 | 0 | 0 | 0 | 0 | 0 | 0 | 0 | 0 | 0 | 0 | 0 | 0 | 0 | 0 | 0 | 0 | 0 | 0 | 0 | 0 |
| Sm152Di | 0 | 0 | 0 | 0 | 0 | 0 | 0 | 0 | 0 | 0.1 | 0.1 | 100 | 0.15 | 0.8 | 0 | 0 | 0 | 0 | 0 | 0 | 0 | 0 | 0 | 0 | 0 | 0 | 0 | 0.55 | 0 | 0 | 0 | 0 | 0 | 0 | 0 | 0 |
| Eu153Di | 0 | 0 | 0 | 0 | 0 | 0 | 0 | 0 | 0 | 0 | 0 | 0 | 100 | 0 | 0 | 0 | 0 | 0 | 0 | 0 | 0 | 0 | 0 | 0 | 0 | 0 | 0 | 0 | 0 | 0 | 0 | 0 | 0 | 0 | 0 | 0 |
| Sm154Di | 0 | 0 | 0 | 0 | 0 | 0 | 0 | 0 | 0 | 0 | 0 | 0 | 0 | 100 | 0 | 0 | 0 | 0 | 0 | 0 | 0 | 0 | 0 | 0 | 0 | 0 | 0 | 0 | 0 | 0 | 0 | 0 | 0 | 0 | 0 | 0 |
| Gd155Di | 0 | 0 | 0 | 0 | 0 | 0 | 0 | 0 | 0 | 0 | 0 | 0 | 0 | 0 | 100 | 0 | 0 | 0 | 0 | 0 | 0 | 0 | 0 | 0 | 0 | 0 | 0 | 0 | 0 | 0 | 3.5 | 0 | 0 | 0 | 0 | 0 |
| Gd156Di | 0 | 0 | 0 | 0 | 0 | 0 | 0 | 0 | 0 | 0 | 0 | 0 | 0 | 0 | 0 | 100 | 0 | 0 | 0 | 0 | 0 | 0 | 0 | 0 | 0 | 0 | 0 | 0 | 0 | 0 | 0 | 0 | 0 | 0 | 0 | 0 |
| Gd157Di | 0 | 0 | 0 | 0 | 0 | 0 | 0 | 0 | 0 | 0 | 0 | 0 | 0 | 0 | 0 | 0 | 100 | 4 | 0 | 0 | 0 | 0 | 0 | 0 | 0 | 0 | 0 | 0 | 0 | 0 | 0 | 0 | 0.4 | 0 | 0 | 0 |
| Gd158Di | 0 | 0 | 0 | 0 | 0 | 0 | 0 | 0 | 0 | 0 | 0 | 0 | 0 | 0 | 0 | 0 | 0 | 100 | 0 | 0 | 0 | 0 | 0 | 0 | 0 | 0 | 0 | 0 | 0 | 0 | 0 | 0 | 0 | 0 | 0 | 0 |
| Tb159Di | 0 | 0 | 0 | 0 | 0 | 0 | 0 | 0 | 0 | 0 | 0 | 0 | 0 | 0 | 0 | 0 | 0 | 0 | 100 | 0 | 0 | 0 | 0 | 0 | 0 | 0 | 0 | 0 | 0 | 0 | 0 | 0 | 0 | 0 | 0.7 | 0 |
| Gd160Di | 0 | 0 | 0 | 0.01 | 0.02 | 0.08 | 0.7 | 0.03 | 0.05 | 0 | 0 | 0.05 | 0 | 0.02 | 0.1 | 0.2 | 0.23 | 0.75 | 0.15 | 100 | 0.3 | 0.25 | 0.05 | 0.07 | 0.02 | 0.02 | 0 | 0 | 0 | 0 | 0 | 0 | 0 | 0 | 0 | 1.5 |
| Dy161Di | 0 | 0 | 0 | 0 | 0 | 0 | 0 | 0 | 0 | 0 | 0 | 0 | 0 | 0 | 0 | 0 | 0 | 0 | 0 | 0 | 100 | 3 | 3 | 1.5 | 0 | 0 | 0 | 0 | 0 | 0 | 0 | 0 | 0 | 0 | 0 | 0 |
| Dy162Di | 0 | 0 | 0 | 0 | 0 | 0 | 0 | 0 | 0 | 0 | 0 | 0 | 0 | 0 | 0 | 0 | 0 | 0 | 0 | 0 | 0 | 100 | 0 | 0 | 0 | 0 | 0 | 0 | 0 | 0 | 0 | 0 | 0 | 0 | 0 | 0 |
| Dy163Di | 0 | 0 | 0 | 0 | 0 | 0 | 0 | 0 | 0 | 0 | 0 | 0 | 0 | 0 | 0 | 0 | 0 | 0 | 0 | 0 | 0 | 0 | 100 | 0 | 0 | 0 | 0 | 0 | 0 | 0 | 0 | 0 | 0 | 0 | 0 | 0 |
| Dy164Di | 0 | 0 | 0 | 0 | 0 | 0 | 0 | 0 | 0 | 0 | 0 | 0 | 0 | 0 | 0 | 0 | 0 | 0 | 0 | 0 | 0 | 0 | 0 | 100 | 0 | 0 | 0 | 0 | 0 | 0 | 0 | 0 | 0 | 0 | 0 | 0 |
| Ho165Di | 0 | 0 | 0 | 0 | 0 | 0 | 0 | 0 | 0 | 0 | 0 | 0 | 0 | 0 | 0 | 0 | 0 | 0 | 0 | 0 | 0 | 0 | 0 | 0 | 100 | 0 | 0 | 0 | 0 | 0 | 0 | 0 | 0 | 0 | 0 | 0 |
| Er166Di | 0 | 0 | 0 | 0 | 0 | 0 | 0 | 0 | 0 | 0 | 0 | 0 | 0 | 0 | 0 | 0 | 0 | 0 | 0 | 0 | 0 | 0 | 0 | 0 | 0 | 100 | 0 | 0 | 0 | 0 | 0 | 0 | 0 | 0 | 0 | 0 |
| Er167Di | 0 | 0 | 0 | 0 | 0 | 0 | 0 | 0 | 0 | 0 | 0 | 0 | 0 | 0 | 0 | 0 | 0 | 0 | 0 | 0 | 0 | 0 | 0 | 0 | 0 | 0 | 100 | 1.7 | 0 | 0 | 0 | 0 | 0 | 0 | 0 | 0 |
| Er168Di | 0 | 0 | 0 | 0 | 0 | 0 | 0 | 0 | 0 | 0 | 0 | 0 | 0 | 0 | 0 | 0 | 0 | 0 | 0 | 0 | 0 | 0 | 0 | 0 | 0 | 0 | 0 | 100 | 0 | 0 | 0 | 0 | 0 | 0 | 0 | 0 |
| Tm169Di | 0 | 0 | 0 | 0 | 0 | 0 | 0 | 0 | 0 | 0 | 0 | 0 | 0 | 0 | 0 | 0 | 0 | 0 | 0 | 0 | 0 | 0 | 0 | 0 | 0 | 0 | 0.1 | 0.05 | 100 | 0.3 | 0.15 | 0 | 0.1 | 0.2 | 0 | 0 |
| Er170Di | 0 | 0 | 0 | 0 | 0 | 0 | 0 | 0 | 0 | 0 | 0 | 0 | 0 | 0 | 0 | 0 | 0 | 0 | 0 | 0 | 0 | 0 | 0 | 0 | 0 | 0 | 0 | 0 | 0 | 100 | 0 | 0 | 0 | 0 | 0 | 0 |
| Yb171Di | 0 | 0 | 0 | 0 | 0 | 0 | 0 | 0 | 0 | 0 | 0 | 0 | 0 | 0 | 0 | 0 | 0 | 0 | 0 | 0 | 0 | 0 | 0 | 0 | 0 | 0 | 0 | 0 | 0 | 0 | 100 | 0 | 0 | 0 | 0 | 0 |
| Yb172Di | 0 | 0 | 0 | 0 | 0 | 0 | 0 | 0 | 0 | 0 | 0 | 0 | 0 | 0 | 0 | 0 | 0 | 0 | 0 | 0 | 0 | 0 | 0 | 0 | 0 | 0 | 0 | 0 | 0 | 0 | 0 | 100 | 0 | 0.5 | 0 | 0 |
| Yb173Di | 0 | 0 | 0 | 0 | 0 | 0 | 0 | 0 | 0 | 0 | 0 | 0 | 0 | 0 | 0 | 0 | 0 | 0 | 0 | 0 | 0 | 0 | 0 | 0 | 0 | 0 | 0 | 0 | 0 | 0 | 0 | 0 | 100 | 0 | 0 | 0 |
| Yb174Di | 0 | 0 | 0 | 0 | 0 | 0 | 0 | 0 | 0 | 0 | 0 | 0 | 0 | 0 | 0 | 0 | 0 | 0 | 0 | 0 | 0 | 0 | 0 | 0 | 0 | 0 | 0 | 0 | 0 | 0 | 0 | 0 | 0 | 100 | 0 | 0 |
| Lu175Di | 0 | 0 | 0 | 0 | 0 | 0 | 0 | 0 | 0 | 0 | 0 | 0 | 0 | 0 | 0 | 0 | 0 | 0 | 0 | 0 | 0 | 0 | 0 | 0 | 0 | 0 | 0 | 0 | 0 | 0 | 0 | 0 | 0 | 0 | 100 | 0.5 |
| Yb176Di | 0 | 0 | 0 | 0 | 0 | 0 | 0 | 0 | 0 | 0 | 0 | 0 | 0 | 0 | 0 | 0 | 0 | 0 | 0 | 0 | 0 | 0 | 0 | 0 | 0 | 0 | 0 | 0 | 0 | 0 | 0 | 0 | 0 | 0 | 0.1 | 100 |

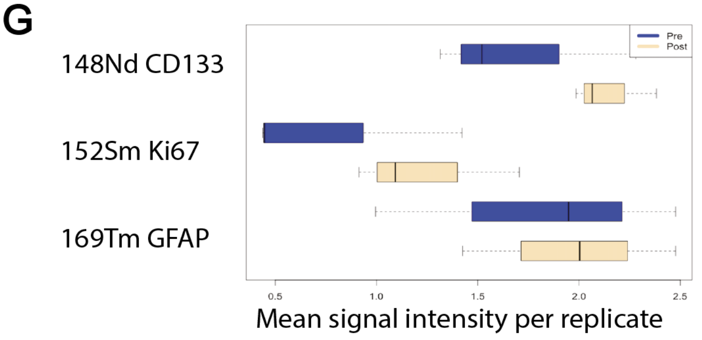

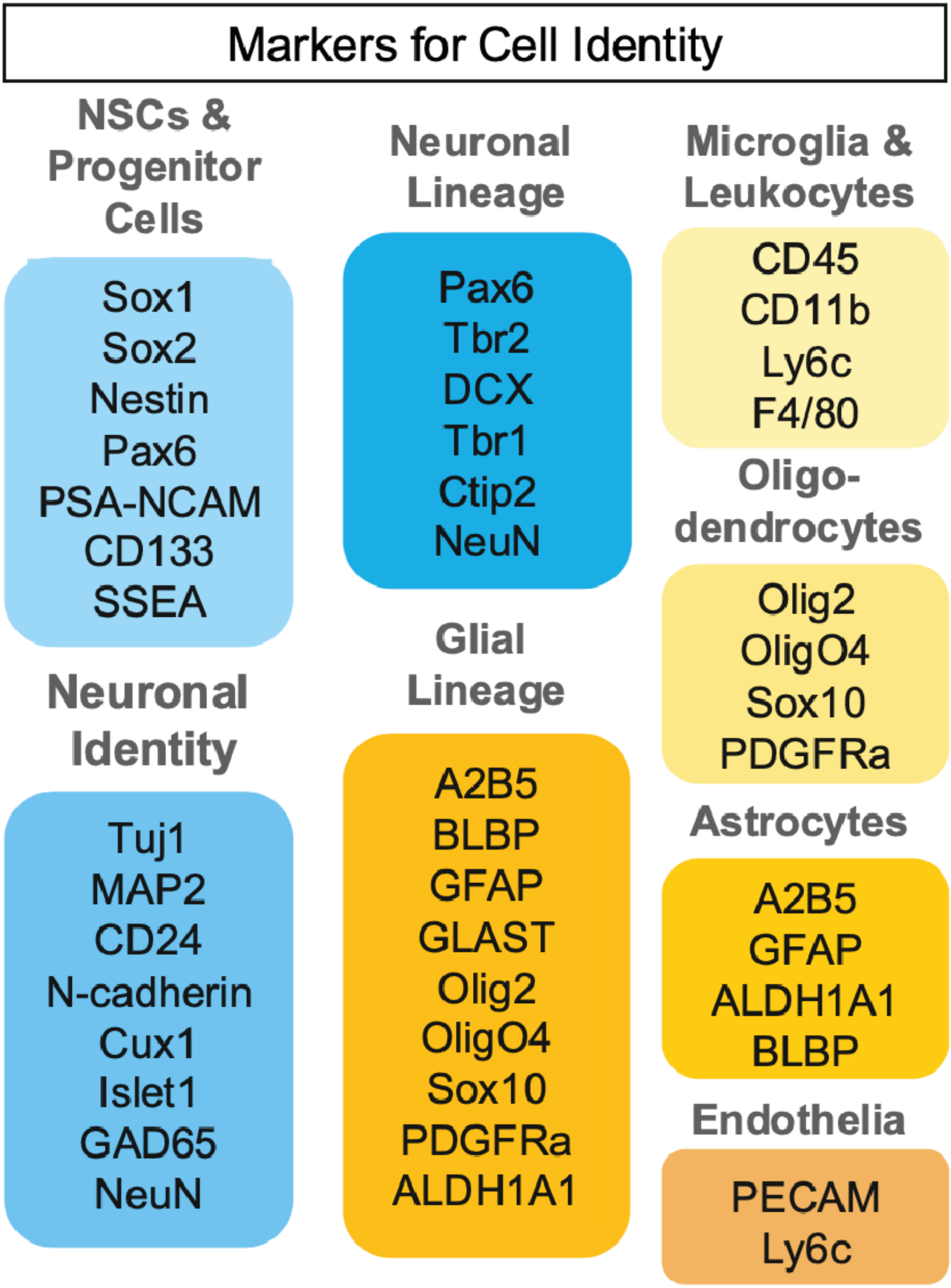
Markers for Cell Identity
NSCs & Progenitor Cells
Sox1
Sox2
Nestin
Pax6
PSA-NCAM
CD133
SSEA
Neuronal Identity
Tuj1
MAP2
CD24
N-cadherin
Cux1
Islet1
GAD65
NeuN
Neuronal Lineage
Pax6
Tbr2
DCX
Tbr1
Ctip2
NeuN
Glial Lineage
A2B5
BLBP
GFAP
GLAST
Olig2
OligO4
Sox10
PDGFRa
ALDH1A1
Microglia & Leukocytes
CD45
CD11b
Ly6c
F4/80
Oligo-dendrocytes
Olig2
OligO4
Sox10
PDGFRa
Astrocytes
A2B5
GFAP
ALDH1A1
BLBP
Endothelia
PECAM
Ly6c

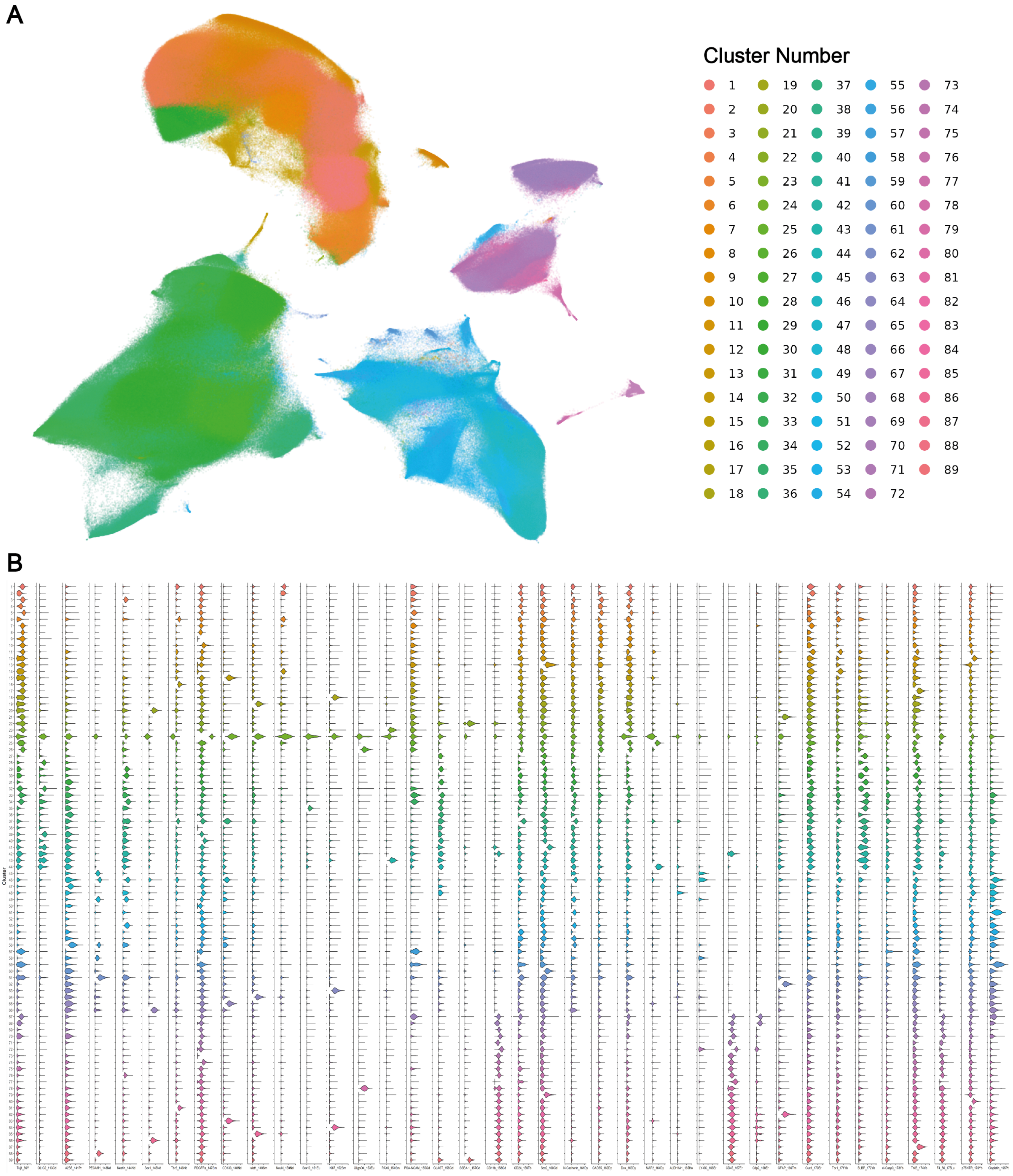
A
Cluster Number
1 2 3 4 5 6 7 8 9 10 11 12 13 14 15 16 17 18
19 20 21 22 23 24 25 26 27 28 29 30 31 32 33 34 35 36
37 38 39 40 41 42 43 44 45 46 47 48 49 50 51 52 53 54
55 56 57 58 59 60 61 62 63 64 65 66 67 68 69 70 71 72
73 74 75 76 77 78 79 80 81 82 83 84 85 86 87 88 89
B
Cluster
Tuj1_89Y
OLIG2_113Cd
A2B5_141Pr
PECAM1_143Nd
Nestin_144Nd
Sox1_145Nd
Tbr2_146Nd
PDGFRa_147Sm
CD133_148Nd
Islet1_149Sm
NeuN_150Nd
Sox10_151Eu
Ki67_152Sm
OligoO4_153Eu
PAX6_154Sm
PSA-NCAM_155Gd
GLAST_156Gd
SSEA-1_157Gd
CD11b_158Gd
CD24_159Tb
Sox2_160Gd
N-Cadherin_161Dy
GAD65_162Dy
Dcx_163Dy
MAP2_164Dy
ALDH1A1_165Ho
LY-6C_166Er
CD45_167Er
Ctip2_168Er
GFAP_169Tm
Cux1_170Er
Tbr1_171Yb
BLBP_172Yb
cl-Casp3_173Yb
TrkB_174Yb
F4_80_175Lu
p75NTR_176Yb
Cisplatin_195Pt

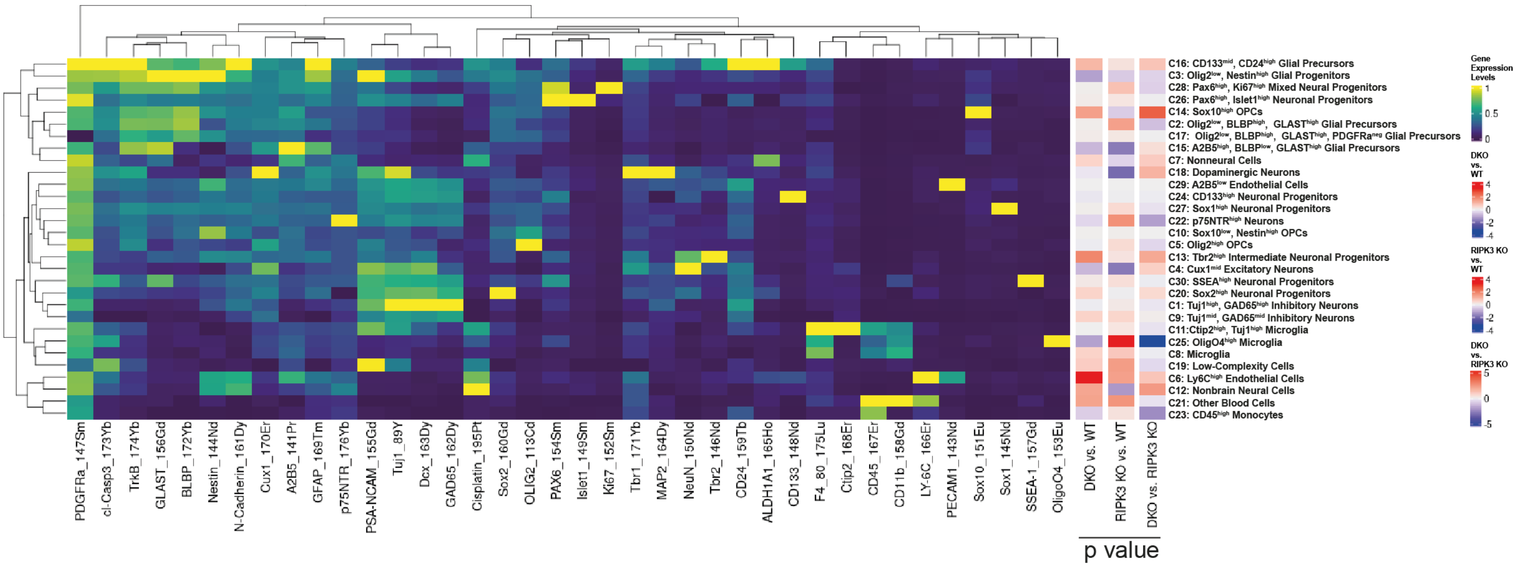
PDGFRa_147Sm
cl-Casp3_173Yb
TrkB_174Yb
GLAST_156Gd
BLBP_172Yb
Nestin_144Nd
N-Cadherin_161Dy
Cux1_170Er
A2B5_141Pr
GFAP_169Tm
p75NTR_176Yb
PSA-NCAM_155Gd
Tuj1_89Y
Dcx_163Dy
GAD65_162Dy
Cisplatin_195Pt
Sox2_160Gd
OLIG2_113Cd
PAX6_154Sm
Islet1_149Sm
Ki67_152Sm
Tbr1_171Yb
MAP2_164Dy
NeuN_150Nd
Tbr2_146Nd
CD24_159Tb
ALDH1A1_165Ho
CD133_148Nd
F4_80_175Lu
Ctip2_168Er
CD45_167Er
CD11b_158Gd
LY-6C_166Er
PECAM1_143Nd
Sox10_151Eu
Sox1_145Nd
SSEA-1_157Gd
OligoO4_153Eu
DKO vs. WT
RIPK3 KO vs. WT
DKO vs. RIPK3 KO
p value
C16: CD133$^{mid}$, CD24$^{high}$ Glial Precursors
C3: Olig2$^{low}$, Nestin$^{high}$ Glial Progenitors
C28: Pax6$^{high}$, Ki67$^{high}$ Mixed Neural Progenitors
C26: Pax6$^{high}$, Islet1$^{high}$ Neuronal Progenitors
C14: Sox10$^{high}$ OPCs
C2: Olig2$^{low}$, BLBP$^{high}$, GLAST$^{high}$ Glial Precursors
C17: Olig2$^{low}$, BLBP$^{high}$, GLAST$^{high}$, PDGFRa$^{neg}$ Glial Precursors
C15: A2B5$^{high}$, BLBP$^{low}$, GLAST$^{high}$ Glial Precursors
C7: Nonneural Cells
C18: Dopaminergic Neurons
C29: A2B5$^{low}$ Endothelial Cells
C24: CD133$^{high}$ Neuronal Progenitors
C27: Sox1$^{high}$ Neuronal Progenitors
C22: p75NTR$^{high}$ Neurons
C10: Sox10$^{low}$, Nestin$^{high}$ OPCs
C5: Olig2$^{high}$ OPCs
C13: Tbr2$^{high}$ Intermediate Neuronal Progenitors
C4: Cux1$^{mid}$ Excitatory Neurons
C30: SSEA$^{high}$ Neuronal Progenitors
C20: Sox2$^{high}$ Neuronal Progenitors
C1: Tuj1$^{high}$, GAD65$^{high}$ Inhibitory Neurons
C9: Tuj1$^{mid}$, GAD65$^{mid}$ Inhibitory Neurons
C11:Ctip2$^{high}$, Tuj1$^{high}$ Microglia
C25: OligO4$^{high}$ Microglia
C8: Microglia
C19: Low-Complexity Cells
C6: Ly6C$^{high}$ Endothelial Cells
C12: Nonbrain Neural Cells
C21: Other Blood Cells
C23: CD45$^{high}$ Monocytes
Gene Expression Levels
1
0.5
0
DKO vs. WT
4
2
0
-2
-4
RIPK3 KO vs. WT
4
2
0
-2
-4
DKO vs. RIPK3 KO
5
0
-5

| Metal | Antibody/Reagent | Full Name(s) | Vendor | Catalog No. | Clone | Concentration |
|---|---|---|---|---|---|---|
| Y89 | TUJ1 | Beta 3-tubulin, Tuj1 | Gift (A. Spano) | - | Tuj1 | 500 ng/mL |
| In113 | Olig2 | Oligodendrocyte transcription factor 2 | Millipore | MABN50 | 211F1.1 | 3000 ng/mL |
| Pr141 | A2B5 | - | Biolegend | 150702 | 105/A2B5 | 50 ng/mL |
| Nd143 | PECAM1 | PECAM-1, Endothelial cell adhesion molecule | Biolegend | 102425 | 390 | 50 ng/mL |
| Nd144 | Nestin | - | R&D Systems | MAB2736 | 307501 | 10 ng/mL |
| Nd145 | Sox1 | SRY-box transcription factor 1 | R&D Systems | AF3369 | Polyclonal | 80 ng/mL |
| Nd146 | Tbr2 | T-box brain gene 2, EOMES, eomesdermin | Thermo Fisher | 14-4875-82 | Dan11mag | 4000 ng/mL |
| Sm147 | PDGFRa | Platelet-derived growth factor alpha | Biolegend | 135902 | APA5 | 100 ng/mL |
| Nd148 | CD133 | Prominin 1 | Biolegend | 141202 | 315-2C11 | 80 ng/mL |
| Sm149 | Islet1 | ISL LIM homeobox 1 | Novus | NBP2-14999 | Polyclonal | 250 ng/mL |
| Nd150 | RBFOX3/NeuN | Neuronal Nuclei | Novus | NBP1-92693 | 1B7 | 200 ng/mL |
| Eu151 | Sox10 | Sex-determining region Y box 10 | Gift (S. Kucenas) | - | Monoclonal | 500 ng/mL |
| Sm152 | Ki67 | MKI67, Marker of proliferation Ki-67 | BD Biosciences | 550609 | B56 | 35 ng/mL |
| Eu153 | OligoO4 | Oligodendrocyte marker 04 | R&D Systems | MAB1326 | O4 | 250 ng/mL |
| Sm154 | Pax6 | Paired box 6 | BD Biosciences | 561462 | O18-1330 | 15 ng/mL |
| Gd155 | PSA-NCAM | Polysialylated-neural cell adhesion molecule | eBioscience | 14-9118-82 | 12E3 | 150 ng/mL |
| Gd156 | GLAST | Excitatory amino acid transporter 1,Glutamate asparate transporter | Novus | NB100-1869 | Polyclonal | 15 ng/mL |
| Gd157 | SSEA-1 | CD15, Stage-specific embryonic antigen | eBioscience | 14-8813-80 | MC-480 | 8 ng/mL |
| Gd158 | CD11b | Integrin aM, Mac-1 | Biolegend | 101249 | M1/70 | 15 ng/mL |
| Tb159 | CD24 | Heat stable antigen | BD Biosciences | 557436 | m1/69 | 10 ng/mL |
| Gd160 | Sox2 | Sex-determining region Y bax 2 | R&D Systems | MAB2018 | 245610 | 3000 ng/mL |
| Dy161 | N-Cadherin | N-cadherin, CD325 | Biolegend | 844702 | 13A9 | 30 ng/mL |
| Dy162 | GAD65 | Glutamic acid decarboxylase 65-kD, glutamate decarboxylase 2 | Biolegend | 844502 | N-CAD65 | 600 ng/mL |
| Dy163 | Dcx | Doublecortin | Thermo Fisher | 481200 | Polyclonal | 500 ng/mL |
| Dy164 | MAP2 | Microtubule-associated protein 2 | Novus | NBP2-25156 | 4H5 | 200 ng/mL |
| Ho165 | ALDH1A1 | Aldehyde dehydrogenase 1A1 | R&D Systems | AF5869 | Polyclonal | 80 ng/mL |
| Er166 | Ly-6C | Lymphocyte antigen 6 complex, locus C | Biolegend | 128002 | HK1.4 | 10 ng/mL |
| Er167 | CD45 | Protein tyrosine phosphatase receptor type C | Fluidigm | 3089005B | 30-F11 | 10 ng/mL |
| Er168 | Ctip2 | COUP-TF-interacting protein 2, Bcl11b | Abcam | ab18465 | 25B6 | 200 ng/mL |
| Tm169 | GFAP | Glial fibrillary acidic protein | BD Biosciences | 556330 | 102 | 7 ng/mL |
| Er170 | Cux1 | Cut-like homeobox 1 | Abcam | ab54583 | 2A10 | 10 ng/mL |
| Yb171 | Tbr1 | T-box brain gene 1 | Abcam | ab31940 | Polyclonal | 1000 ng/mL |
| Yb172 | BLBP | Brain lipid-binding protein, fatty acid binding protein 7, FABP7 | Gift (C. Birchmeier | - | Polyclonal | 1000 ng/mL |
| Yb173 | cl-Casp3 | cleaved Caspase-3 | BD Biosciences | 559565 | C92-605 | 2000 ng/mL |
| Yb174 | TrkB | Neurotrophic tyrosine kinase receptor type 2 | Thermo Fisher | AF1494 | Polyclonal | 300 ng/mL |
| Lu175 | F4/80 | EMR1, Ly-71 | Biolegend | 123101 | BM8 | 5 ng/mL |
| Yb176 | p75NTR | P75 neurotrophic receptor, TNF receptor 16 | R&D Systems | AF1157 | Polyclonal | 150 ng/mL |
| Ir191/193 | Intercalator | Cell-ID Intercalator-Ir | Fluidigm | 201192A | - | 1:5000 dilution |
| Pt194/195/198 | Cisplatin | Cisplatin | Sigma Aldrich | P4394 | - | 5 µM |

# Figure Legends

## Supplementary Fig. S1 Cell Death, Proliferation, and Abundance in the Developing Telencephalon.

(A) Representative flow cytometry plots showing distinct cell populations based on CC3 and Cisplatin expression patterns during development: $CC3^{+}Cisplatin^{-}$ (early apoptosis), $CC3^{-}Cisplatin^{+}$ (non-apoptotic death), and $CC3^{+}Cisplatin^{+}$ (late-stage apoptosis or mixed death mechanisms).

(B) Violin plots showing marker expression distribution across 23 cell clusters (rows) for 37 protein markers (columns).

(C) Analysis of neuronal cluster dynamics during development. Top: UMAP visualizations highlighting individual clusters categorized into immature and mature neuronal populations. Bottom: Temporal profiles from E13 to P4 showing cluster abundance (gray), percentage of $CC3^{+}$ cells (orange), $Cisplatin^{+}$ cells (green), and $Ki67^{+}$ cells (blue). Points represent individual replicates, and solid lines show Loess curve fitting of the data.

## Supplementary Fig. S2 Analysis of Nonneuronal Subpopulations Dynamics during Development.

Top: UMAP visualizations highlighting individual clusters categorized into immature and mature nonneuronal populations. Bottom: Temporal profiles from E13 to P4 showing cluster abundance (gray), percentage of $CC3^{+}$ cells (orange), $Cisplatin^{+}$ cells (green), and $Ki67^{+}$ cells (blue). Points represent individual replicates, and solid lines show Loess curve fitting of the data.

## Supplementary Fig. S3 Cisplatin Gating Workflow and Robustness Analysis.

(A) Density plots of $^{195}$Pt-Cisplatin signal for all 22 telencephalic samples (E13–P4). One single sample (RUN1_BCSet1_E14; red highlight) showed an elevated baseline.

(B) Dot plots ($^{191}$Ir-Intercalator vs $^{195}$Pt-Cisplatin) illustrating the standard rectangular live-cell gate (upper panel, representative normal sample) and the proportional gate used for the outlier (lower panel).

(C) Gate-placement sensitivity test. Left: dot plots of pooled events from 21 standard-gated samples with three Cisplatin gates—high stringency (≥350 counts), medium (≥100), and low (≥30)—outlined in red. Right: LOESS curves ± 95 % CI showing Cisplatin$^{+}$ Cells% versus developmental age under each gate; trajectories are super-imposable, indicating gate independence.

(D–F) Temporal profiles of Cisplatin$^{+}$ Cell% for all clusters across the full cohort (n = 22). Points represent individual replicates, and solid lines show Loess curve fitting of the data.

## Supplementary Fig. S4 Antibody Testing of Cleaved Caspase-8 (CC8) by IHC during Telencephalic Development.

Representative immunofluorescence images of mouse brain sections from E13.5 to P0. Sections were stained with DAPI (blue), cleaved Caspase-3 (CC3) (red, Cell Signaling #9661S), and two different CC8 antibodies: Cleaved Caspase-8 (Asp387) (D5B2) XP® Rabbit mAb, Cell Signaling #8592S and Cleaved Caspase-8 (Asp384) (11G10) Mouse mAb, Cell Signaling #9748S. Multiple developmental timepoints were examined to assess CC8 antibody specificity and optimize staining conditions. Scale bar: 50 µm.

## Supplementary Fig. S5 Antibody Testing of pRIPK3 and pMLKL by IHC Using WT, RIPK3 KO and DKO Brain Sections.

(A) Representative immunofluorescence images of mouse brain sections from WT and DKO mice. Sections were stained with DAPI (blue) and pMLKL (Phospho-MLKL (Ser345) (D6E3G) Rabbit mAb, Cell Signaling #37333S, red).

(B) Representative immunofluorescence images of mouse brain sections from WT, RIPK3 KO and DKO mice. Sections were stained with DAPI (blue) and pRIPK3 (Phospho-RIP3 (Thr231/Ser232) Antibody (Mouse Specific), Cell Signaling #57220S, red). Scale bar: 50 μm.

## Supplementary Fig. S6 RNAscope Detection of Casp8 and Ripk3 mRNA in mouse cortex

(A) Quantification of spatial proximity at postnatal day 4 (P4). The box-and-whisker plot shows the percentage of Casp8 puncta who lies ⩽ 6 pixels (~1.2 μm) from the nearest Ripk3 punctum in wild-type telencephalon (n = 3). Dots represent individual mice.

(B) Representative images of RNAscope in situ hybridization for caspase-8 (magenta) and ripk3 (yellow) mRNA in a 6-month mouse cortex. Images were captured at both 20× and 40× magnification to show both broad distribution patterns and cellular-level expression details. Scale bar: 50 μm.

## Supplementary Fig. S7 Sex-specific analysis in P4 telencephalon.

(A) Quantification of total cell numbers in the telencephalon of P4 male (M) and female (F) mice across genotypes (WT, RIPK3 KO, and DKO; n = 6 per group).

(B) Quantification of $CC3^+$, $Cisplatin^+$, and $Ki67^+$ cells% in the telencephalon of P4 male (M) and female (F) mice across genotypes (WT, RIPK3 KO, and DKO; n = 6 per group). Data presented as box plots where the middle line represents the median, the box represents the interquartile range (IQR), and the whiskers extend to 1.5 times the IQR. Individual data points are shown as dots. Statistical differences between groups are denoted by asterisks.

## Supplementary Fig. S8 Pre-processing Workflow for Mass Cytometry Data

(A) Raw data normalization using calibration beads, showing signal intensities before and after normalization.

(B) Debarcoding analysis of normalized data across three barcode sets, with event count distributions (top) and yield percentages (bottom) for each sample.

(C-E) Sequential gating strategy for single-cell isolation: barcode stringency (87.98%), event-centered mass cytometer isolation (94.16%), and bead removal (98.64%). Gates shown in red with percentages indicating cell yield.

(F) Signal compensation matrix used to correct for metal isotope spillover between channels.

(G) Comparison of mean signal intensities before (blue) and after (yellow) batch correction for CD133, Ki67, and GFAP. Only markers with mean signal variance > 0.01 underwent batch correction; all other markers showed minimal variance and remained uncorrected.

## Supplementary Fig. S9 Cell Type-specific Markers Used for Mass Cytometry Analysis.

Overview of protein markers used to define distinct cell populations: neural stem cells and progenitors (blue, top left), general neuronal identity (blue, bottom left), neuronal lineage (blue, top middle), glial lineages (yellow, bottom middle), and nonneuronal cell types including microglia, oligodendrocytes, astrocytes, and endothelial cells (orange, right).

## Supplementary Fig. S10 Refined Clustering Analysis of Mass Cytometry Data.

(A) UMAP visualization showing 89 distinct cell clusters identified by secondary Leiden clustering analysis. Clusters are color-coded and numbered (1-89) as shown in the legend.

(B) Violin plots depicting marker expression distribution across all 89 clusters (rows) for 37 protein markers (columns).

## Supplementary Fig. S11 Genotype-Dependent Abundance Differences Across Initial Clusters in the Developing Telencephalon.

Heatmap showing marker gene expression across 30 identified cell clusters (rows) from the initial cluster with corresponding p-values comparing relative abundance between genotypes (DKO vs. WT, RIPK3 KO vs. WT and DKO vs. RIPK3 KO). The main heatmap shows gene expression levels where yellow indicates higher expression and blue indicates lower expression (scale shown on right). The adjacent p-value heatmap (3 right columns) displays statistical significance of abundance differences between genotypes (DKO vs. WT, RIPK3 KO vs. WT and DKO vs. RIPK3 KO), where red indicates higher relative abundance and blue indicates lower relative abundance (scale shown on right). For all quantifications, statistical differences were calculated using Student's t-test.

## Supplementary Table 1. Mass Cytometry Antibody Panel.

Comprehensive list of antibodies used in mass cytometry analysis showing metal isotope labels (Metal), antibody name (Antibody/Reagent), and full protein names or descriptions (Full Name(s)), vendor information (Vendor), catalog numbers (Catalog No.), clone identifiers (Clone), working concentrations (concentration).
